# Experimental community coalescence sheds light on microbial interactions in soil and restores impaired functions

**DOI:** 10.1186/s40168-023-01480-7

**Published:** 2023-03-04

**Authors:** Sarah Huet, Sana Romdhane, Marie-Christine Breuil, David Bru, Arnaud Mounier, Ayme Spor, Laurent Philippot

**Affiliations:** University Bourgogne Franche-Comte, INRAE, Institut Agro Dijon, Agroecologie Department, 17 rue de Sully, Dijon, 21000 France

**Keywords:** Microbial interactions, Community manipulation, Coalescence, Restoration, Soil functions, Density-dependent interactions

## Abstract

**Background:**

Microbes typically live in communities where individuals can interact with each other in numerous ways. However, knowledge on the importance of these interactions is limited and derives mainly from studies using a limited number of species grown in coculture. Here, we manipulated soil microbial communities to assess the contribution of interactions between microorganisms for assembly of the soil microbiome.

**Results:**

By combining experimental removal (taxa depletion in the community) and coalescence (mixing of manipulated and control communities) approaches, we demonstrated that interactions between microorganisms can play a key role in determining their fitness during soil recolonization. The coalescence approach not only revealed the importance of density-dependent interactions in microbial community assembly but also allowed to restore partly or fully community diversity and soil functions. Microbial community manipulation resulted in shifts in both inorganic nitrogen pools and soil pH, which were related to the proportion of ammonia-oxidizing bacteria.

**Conclusions:**

Our work provides new insights into the understanding of the importance of microbial interactions in soil. Our top-down approach combining removal and coalescence manipulation also allowed linking community structure and ecosystem functions. Furthermore, these results highlight the potential of manipulating microbial communities for the restoration of soil ecosystems.

Video Abstract

**Supplementary Information:**

The online version contains supplementary material available at 10.1186/s40168-023-01480-7.

## Background

Microbes form complex and highly diverse communities that have an essential role in ecosystem functioning [1, 2]. In the last few decades, evidence has arisen that these functions performed by microbial communities are intrinsically related to their diversity and composition [3–5]. While microorganisms can interact with each other in numerous ways [6–8], only limited insights exist about the contribution of such biotic interactions to the assembly and composition of microbial communities. They are often obtained from simplified microbial systems in which pairwise interactions between strains are monitored [9–11]. Competition in particular has been suggested as the dominant type of interaction among microbial species [10, 12]. However, because of the simplicity of these systems, it is uncertain if these reductionist approaches can live up to their promise of providing a better understanding of interactions between microbes in their real habitat. For example, pairwise species interactions often fail to predict interactions in more complex systems likely due to higher-order interactions, which arise in the presence of additional species [13]. Therefore, a better understanding of interactions between microorganisms in complex communities is needed to better predict microbial community assembly, which is key to rationally engineer or manipulate these complex communities for our own ends.

Community coalescence is a recently introduced concept describing the encounter of previously separate microbial communities [14]. During these encounter events, novel interactions are generated and Rillig et al. [14] therefore proposed that an explicit consideration of coalescence could help better understand the complexity of microbial assemblages as well as the importance of microbial interactions. Since even the most degraded ecosystems are unlikely to be sterile, the coalescence of microbial communities has also recently been investigated in relation to ecological restoration of degraded ecosystems [15]. Thus, due to their critical roles in biogeochemical cycling, microbial communities are now seen as a system component to be manipulated for promoting the recovery of ecosystems [16]. For example, Wubs et al. [17] tested the application of soil inocula in the field and showed coalescence could steer the soil community and promote nature restoration.

**Fig. 1:**
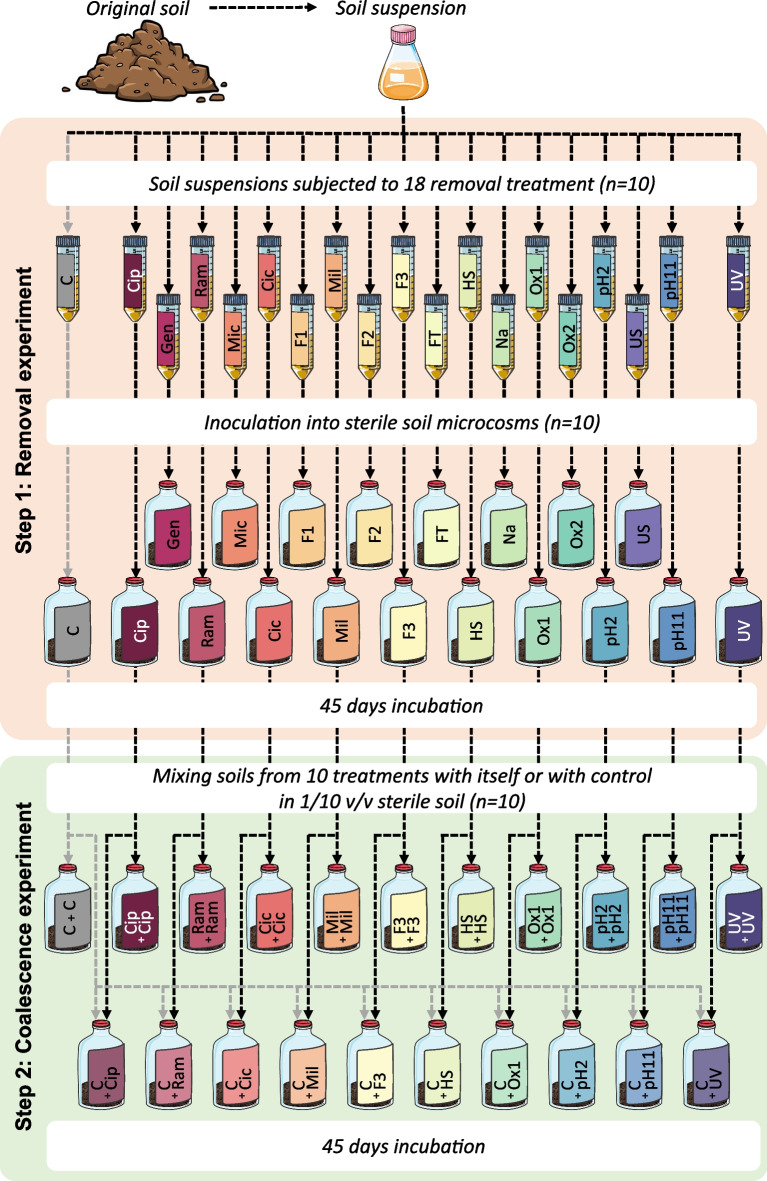
Schematic illustration of the experimental design. The soil microbial community was manipulated using a top-down approach in two steps. Step 1 consisted in a removal approach during which soil suspensions were either subjected to one out of 18 removal treatments (R, *n*=10) to deplete various microbial groups or were not treated (control, C, *n*=10). Step 2 consisted in a coalescence approach during which soils from 10 removal treatments were either mixed with themselves (R+R) or with the soil from the non-treated microcosms (R+C) and non-treated soil was mixed with itself (C+C) in 1/10 v/v sterile soil (*n*=10). Values in parentheses indicate the number of biological replicates

Here we used a two-step, top-down manipulation of soil microbial communities based on removal and coalescence approaches to assess the importance of interactions between microorganisms for soil microbial community assembly and functions (Fig. 1). As proposed by Rillig et al. (2016, Front. Microbiol.), community coalescence in our work stands for the mixing of soils containing manipulated and non-manipulated microbial communities. For this purpose, we first subjected a soil microbial community to 18 different removal treatments (Table 1) before reinoculation in its native, but sterilized, soil to allow the different populations to assemble during recolonization. We then applied a generalized linear mixed model to identify the OTUs with significant changes in relative abundance (used as a proxy of the relative fitness) in the manipulated communities compared to the non-treated control community after 45 days of incubation. To test the hypothesis that depletion of competitors by the removal treatments was behind the observed increase in the relative abundance of a large fraction of the dominant prokaryotic and eukaryotic OTUs across treatments, we then used a coalescence approach by mixing the depleted and control communities and postulated that it would re-establish the initial interactions. Finally, to assess to which extent treatment-induced changes in soil properties and functions were related to shifts in microbial communities, we applied a multi-omics integrative analysis, which supported the usage of removal and coalescence experiments for validating structure–function associations [18].

**Table 1:** Description of the removal treatments applied to soil suspension in Step 1 and the corresponding abbreviations used in figures and text

Treatment	Abbreviation	Description
**Control**	C	Not treated soil suspension
**Ciprofloxacin**	Cip	Antibiotic, 66.67 μg/ml for 5h; wash (×3)
**Gentamicin**	Gen	Antibiotic, 69.44 μg/ml for 5h; wash (×3)
**Ramoplanin**	Ram	Antibiotic, 69.44 μg/ml for 5h; wash (×3)
**Ciclopirox**	Cic	Fungicide, 200 μg/ml for 5h; wash (×3)
**Micafungin**	Mic	Fungicide, 66.67 μg/ml for 5h; wash (×3)
**Miltefosin**	Mil	Protisticide, 69.44 μg/ml for 5h; wash (×3)
**Filtration 1**	F1	Fraction > 5 μm (5-μm filter)
**Filtration 2**	F2	5 μm > fraction > 3 μm (3-μm filter)
**Filtration 3**	F3	3 μm > fraction > 1 μm (1-μm filter)
**Freeze-Thaw**	FT	6 x (15 min at −80°C then 15 min at +30°C)
**Heat shock**	HS	0°C for 5 min/70°C for 15 min/0°C for 5 min
**Osmotic**	Na	NaCl, 0.1 g/ml for 2 h; wash (×3)
**Oxidative 1**	Ox1	H_2_O_2_ 50 mM for 2 h; wash (×3)
**Oxidative 2**	Ox2	H_2_O_2_ 25 mM for 2 h; wash (×3)
**Alkaline**	pH11	0.5 mL ammoniac 20% at 1 M for 2 h; wash (×3)
**Acid**	pH2	1 mL malic acid at 1 M for 2 h; wash (×3)
**Sonication**	US	9 cycles of 30 s ultrasounds (Vibracell VC-500, 20 kHz) and 30 s rest
**UV**	UV	2-h exposure

## Methods

### Experimental design

A sandy loam soil (6.9 % clay, 19 % loam, 74.1 % sand, pH 5.5, and C and N content 14.7 g kg^-1^ and 1.19 g kg^-1^ dry soil, respectively) recognized as a reference [19] was collected at the CNRS Ecology and Environment Institute research station CEREEP, France (48° 16′ 59.5′′ N, 2° 40′ 18.5′′ E) and sieved through 4 mm. In the first step, the soil microbial community was manipulated by applying 18 different removal treatments (Fig. 1 and Table 1). Briefly, each removal treatment was applied independently to 1:10 suspensions of the sieved soil (*n*=10) and 14.2 mL of the treated suspension was inoculated into 147 mL plasma flasks containing 50 g of γ-sterilized soil (2 times 35 kGy; Conservatome, Dagneux, France). Ten replicate microcosms inoculated with non-treated soil suspensions were used as controls. All of the 190 soil microcosms were closed with sterile lids allowing gas exchange and incubated at 23 °C at a soil moisture ranging between 60 and 80% of the soil water-holding capacity for 45 days. After incubation, soil microcosms and the original soils were used for subsequent analyses. In a second step, ten removal treatments were selected for the coalescence experiment (Fig. 1). Those ten treatments were selected to represent the variety of the Step 1 removal treatment types: antibiotics (Cip and Ram), fungicide (Cic), protisticide (Mil), filtration (F3), heatshock (HS), oxidative stress (Ox1), pH (pH2 and pH11), and shortwave (UV). For this purpose, 2.5 g of soil from a removal treatment microcosm (R) was thoroughly mixed with 2.5 g of the non-treated control soil (C) into 45 g of sterile soil microcosms, which corresponds to the coalescence treatment (R+C). Soils from removal treatments and the control were also mixed separately with 45 g of sterile soil to obtain the self-mixed removal treatments (R+R) and the self-mixed control (C+C), respectively. Soil microcosms from Step 2 were also replicated 10 times and incubated under the same condition as Step 1 for 45 days.

### Soil pH, inorganic nitrogen pools, and carbon cycle related activities

Impacts of microbial community manipulations on soil functions and properties were assessed using 5 replicate soil samples from each treatment. Inorganic nitrogen pools and respiration rates of various C substrates were used as indicators of the activity of the microbial guilds involved in N and C cycling, respectively. Soil pH was measured in water (ISO 10390:2005). Soil mineral nitrogen (NO_3_^-^ and NH_4_^+^) was extracted from 10 g fresh soil using 50 mL of KCl (1 M), then shaken at 80 rpm for 1 h at room temperature, filtered and quantified by colorimetry (ISO standard 14256-2). Microbial respiration rates were measured according to the MicroResp method [20] using a plate reader (TECAN Infinite® M200 Pro) for three different C substrates: D-(-)fructose, L-arginine, and gallic acid.

### Assessment of microbial community composition and diversity

DNA was extracted from 400 samples (ten original soil samples, 190 Step 1 microcosms, and 210 Step 2 microcosms, *n*=10) using the DNeasy PowerSoil-htp 96 well DNA isolation kit (Qiagen, France) according to the manufacturer’s instructions. To generate amplicons, a 2-step PCR approach was used according to Berry et al. [21]. The V3–V4 hypervariable region of the 16S rRNA gene and the V4 hypervariable region of the 18S rRNA gene were amplified using the 341F (5’-CCTACGGGRSGCAGCAG-3’) and 805R (5’-GACTACCAGGGTATCTAAT-3’) and the EK-565F (5′-GCAGTTAAAAAGCTCGTAGT-3′) and 18S-EUK-1134-R–UnonMet (5′-TTTAAGTTTCAGCCTTGCG-5′) primers, respectively. The amplicon size was checked with 2% agarose gel, and the DNA concentration was estimated using Quant-IT™ dsDNA HS Assay kit (Invitrogen™, Carlsbad, CA, USA). Final PCR products were purified, and their concentration were normalized using the SequalPrep Normalization plate kit (Invitrogen™, Carlsbad, CA, USA). Sequencing was performed on MiSeq (Illumina, 2 x 250 bp and 2 x 300 bp for 16S and 18S rRNA amplicons, respectively) using the MiSeq reagent kit v2. Demultiplexing and trimming of Illumina adaptors and barcodes was done with Illumina MiSeq Reporter software (version 2.5.1.3). Sequence data from the 400 soil samples were analyzed using an in-house developed Python pipeline (available upon request). Briefly, 16S rRNA and 18S rRNA gene sequences were assembled using PEAR (version 0.9.8) [22] with default settings. Further quality checks were conducted using the QIIME pipeline (version 1.9.1) [23] and short sequences were removed (< 400 bp for 16S and < 475 bp for 18S). Reference based and de novo chimera detection, as well as OTUs clustering were performed using VSEARCH (version 2.14.2) [24] and the adequate reference databases (SILVA’ representative set of sequences version 138.1/2020 [25] for 16S rRNA and the PR2 sequence database version 4.11.1 [26] for 18S rRNA). The identity thresholds were set at 94% for 16S based on replicate sequencing of a bacterial mock community [27] and 97% for 18S. Representative sequences for each OTU were aligned using Infernal (version 1.1.3) [28], and phylogenetic trees were construct using FastTree (version 2.1.11) [29]. Taxonomy was assigned using UCLUST (from USEARCH version 11) [30] and the SILVA database (version 138.1/2020) [25] and the PR2 database (version 4.11.1) for the 16S and 18S rRNA sequences, respectively.

### Quantification of microbial communities

The abundances of total bacterial and fungal microbial communities as well as that of N-cycle microbial guilds were estimated by real-time quantitative PCR (qPCR) assays. For each treatment, we used five equimolar mixtures prepared from pairs of the 10 DNA extracts. Total bacterial and fungal communities were quantified using 16S rRNA and ITS primers as described by Muyzer et al. [31] and White et al. [32], respectively. Abundances of N-cycle microbial guilds were estimated using the *amoA* gene to quantify bacterial (AOB) and archaeal (AOA) ammonia-oxidizers, the *nirK* and *nirS* genes to quantify denitrifiers [33] and the *nifH* gene for the diazotrophs [34]. Real-time qPCR assays were carried out in a ViiA7 (Life Technologies, USA) with a Takyon Master Mix (Eurogentec, France) as previously described [33]. PCR efficiency for the different assays, each one performed in two independent runs, ranged from 79.32 to 104.68 %. No template controls gave null or negligible values. The PCR inhibitor’s presence was tested by mixing soil DNA extracts with either control plasmid DNA (pGEM-T Easy Vector, Promega, France) or water. No inhibition was detected in any case.

### Statistical analysis

Statistical analyses were conducted using R statistical software version 4.0.3 [35]. Differences between treatments in gene copy abundances (16S rRNA, ITS, *amoA*, *nirK*, *nirS,* and *nifH*), ammonium and nitrate concentrations, pH, microbial respiration measurements (*n* = 5), and the microbial α-diversity indices (*n*=10) were tested using ANOVAs followed by Tukey’s honestly significant difference (HSD) test (*p* value ≤ 0.05) using the agricolae package [36]. Normality and homogeneity of the residual distribution were verified, and log-transformations were performed for gene copy abundances. Prokaryotic and eukaryotic α-diversity metrics (i.e., observed species, Simpson’s reciprocal, Shannon and Faith’s Phylogenetic Diversity PD [37]) and Weighted Unifrac distance between samples [38] were calculated based on rarefied OTU tables (means of 16,215 and 15,399 reads per sample rarefied at 9000 sequences and 8000 sequences per sample for the 16S rRNA and 18S rRNA, respectively). We also performed principal components analyses (PCoA) and permutated analysis of variance using the ordin and adonis function of the vegan package (version 2.6-2), respectively [39] based on Weighted Unifrac distance matrix to detect changes in the microbial community structure. We implemented pairwise comparisons between treatment using the pairwise.adonis function from the pairwiseAdonis package (version 0.4). As Weighted Unifrac distances range from zero for similar samples to one for dissimilar samples, we calculated the similarity as one minus the distance.

#### Identification of OTUs differentially abundant in treatments

As zero counts in sequencing datasets can inflate the number of false positive for the differential abundance analysis, we first filtered out low-abundance OTUs by keeping those that (i) represented > 0.5% of the sequences in at least one sample and (ii) were found in at least 60% of replicates for any given treatment, which resulted in 515 and 439 OTUs for the 16S rRNA and the 18S rRNA, respectively. These most abundant OTUs were used to build pruned trees using the ape package [40] and were visualized using the Interactive Tree of Life (iTOL) webserver [41].

To estimate which OTUs significantly differ in relative abundance between the manipulated and control communities, we used a generalized linear mixed model for each of the experiment steps. Such model combines a generalized linear model, which allow to infer linear regression from data that does not follow a normal distribution as abundance data typically follow a Poisson distribution, with a mixed model, which contain both fixed effects (treatment effects) and random effects (sampling effects). Considering that an OTU of abundance *Y*, in any *k* replicates of any *i* Step 1 treatment or *ij* Step 2 treatment, follows a Poisson law of parameter Λ as $\textrm{Y}\sim \mathcal{P}\left(\varLambda \right)$, we used the following models for the Step 1 and Step 2, respectively:

1 $$\log \left({\Lambda}_{ik}\right)={o}_{ik}+\upmu +{\upalpha}_i+{Z}_{ik},\kern0.5em {Z_{ik}}_{1\le j\le 10}\ \textrm{iid}\sim \mathcal{N}\left(0,{\sigma}^2\right)$$

2 $$\log \left({\Lambda}_{ij k}\right)={o}_{ij k}+\upmu +{\beta}_{ij}+{C}_{ij}+{Z}_{ij k},\kern0.5em {Z_{ij k}}_{1\le j\le 10}\ \textrm{iid}\sim \mathcal{N}\left(0,{\sigma}^2\right)$$

where *i* = {1, …, 19} represents the Step 1 treatments, *j* = {1, 2} represents the Step 2 self-mixed or coalescence treatment respectively, *k* = {1, …, 10} represents the replicates, *o* is the offset for each sample calculated as the log of the sample read sum, *α* is the effect of the Step 1 treatments, *Z* is the random sampling effect modeling the data overdispersion, *β* is the effect of the Step 2 treatment, and *C* is the mixed effect modeling the degree of kinship between the Step 2 samples. The analysis was performed using the glmer function of the *lme4* package (version 1.1-27). Subsequently, we performed multiple pairwise comparisons with the emmeans function of the *emmeans* package (version 1.6.1) and post hoc Tukey tests for *p* value adjustment. We selected pairwise comparisons: (i) between each removal treatment and the Step 1 control (R versus C in Step 1), (ii) between each self-mixed removal treatment and the Step 2 control (R+R versus C+C in Step 2), (iii) between each coalescence treatments and its respective self-mixed treatment (R+C versus R+R in Step 2), and (iv) between each coalescence treatment and the Step 2 control (R+C versus C+C in Step 2). Significant comparisons resulted from Tukey test *p* value (*p* value ≤ 0.05). A loglikelihood ratio test was applied when the OTU had a null abundance in one treatment and a median abundance higher or equal to 5 in the other treatment (see code available online).

#### Inference of co-occurrence networks

Networks were constructed based on the most abundant OTU count data (low-abundance OTUs filtered out) using all samples from the cleaned dataset except original soil samples (389 and 386 samples for 16S and 18S, respectively). Networks were inferred using a sparse multivariate Poisson log-normal (PLN) model with a latent Gaussian layer and an observed Poisson layer using the PLNmodels package [42] with an offset corresponding to the number of reads in each sample. The best network was selected using a Stability Approach to Regularization Selection (StARS) [43], retaining significant partial correlations (*ρ*) that represent the degree of association between two variables (OTU abundance) from which the effect of a set of other controlling variables is removed (sequencing depth). For visualization purpose, only partial correlations with an absolute value higher than 0.08 (|*ρ*| > 0.08) were shown using Cytoscape [44].

#### Multivariate integration to identify correlation between OTUs and variables

To identify significantly correlated variables (Pearson’s correlation |*r*|> 0.6) among 16S rRNA sequences (low-abundant OTUs filtered out), gene copy abundances (16S rRNA, *amoA*, *nirK*, *nirS*, *nifH*, and ITS), inorganic N-pools, microbial respiration rates, and soil pH, we used DIABLO (Data Integration Analysis for Biomarker discovery using a Latent component method for Omics studies) from the mixOmics package [45, 46]. This approach is a supervised analysis for the integration of multiple data sets based on a multiblock sparse partial least square discriminant analysis (Multiblock sPLS-DA). The training set used is described in the code available online.

## Results

### Alteration in soil microbiome diversity and composition after the removal treatments

**Fig. 2:**
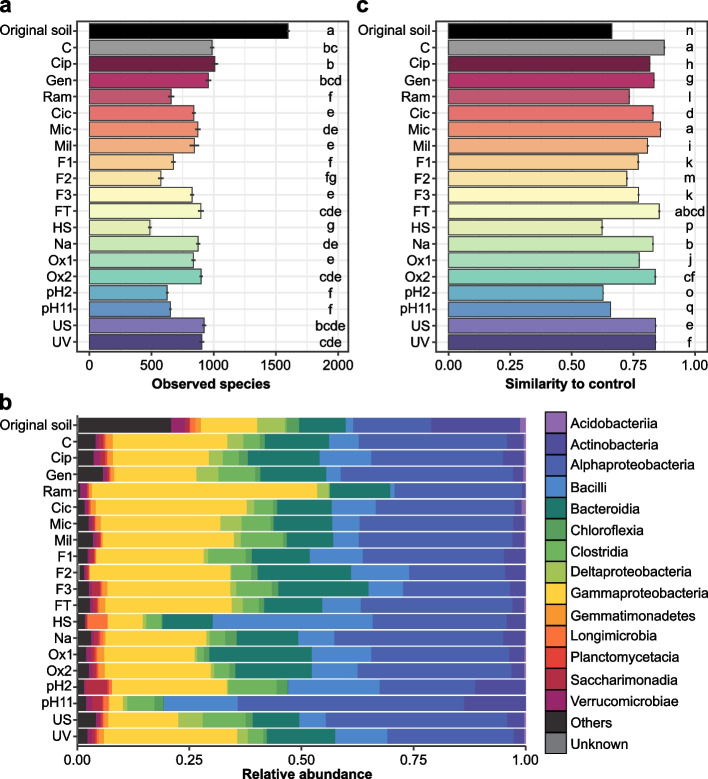
Structure and composition of the prokaryotic communities in the original soil and after Step 1. **a** Number of observed species (mean ± s.e.). The letters indicate significantly different statistical groups (Tukey’s test, *p* value ≤ 0.05). **b** Relative abundances of the fourteen most abundant classes of prokaryotic community. **c** Similarity between the control samples and between the control and either the original soil or the removal treatment, based on the Weighted UniFrac distances (mean ± s.e.). The letters indicate significantly different statistical groups (Adonis pairwise comparison, Benjamini-Hochberg corrected *p* value ≤ 0.05)

In Step 1, we used 18 different removal treatments (Table 1) with antibiotic, fungicide, protisticide, and filtration being selective removal treatments while the others were more general. Quantification of 16S and ITS gene copy numbers indicated that after the 18 removal treatments, the inoculated microbial communities reached the same densities as in the control soil, except for the heat shock and pH2 treatments for both bacteria and fungi and the F3 filtration treatment for the fungi only (post hoc Tukey *p* value ≤ 0.05; Supplementary Figure 1a,b). Ten to 17 of the 18 depletion treatments led to a significant decrease in prokaryotic *α*-diversity compared to the control, depending on the indices (post hoc Tukey *p* value ≤ 0.05; Fig. 2a and Supplementary Figure 2a,b). Overall, the ramoplanin, F2 filtration, heat shock, pH2, and pH11 treatments caused the largest declines in prokaryotic diversity as illustrated by losses up to 50.77% of the observed species compared to the control community (post hoc Tukey *p* value ≤ 0.05; Fig. 2a). The depleted microbial taxa differed between treatments with, for example, the ramoplanin treatment affecting mostly Actinobacteria, Bacilli, and Clostridia while mostly γ-Proteobacteria were depleted by the heat shock treatment (Fig. 2b). In contrast, the F2 and F3 filtration, heat shock, and pH11 treatments had the strongest effect on the eukaryotic community diversity and composition (post hoc Tukey *p* value ≤ 0.05; Supplementary Figures 2c,d and 3a,b). Changes in similarity between manipulated and control communities were consistent with the *α*-diversity results (Adonis pairwise comparison, Benjamini-Hochberg corrected *p* value ≤ 0.05; Fig. 2c, Supplementary Figure 3c and 4).

Next, we used a generalized linear mixed model to identify among the dominant OTUs those exhibiting a differential abundance between the removal treatments (*R*) and the control (*C*). We hypothesized that depletion of some taxa would allow their direct or indirect competitors to thrive, therefore increasing their relative abundance. Accordingly, we found that depletion of various microbial taxa positively affected the relative abundance of 245 prokaryotic and 90 eukaryotic OTUs across treatments (R>C, i.e., significantly higher increase in relative abundance of a given OTU in the manipulated community after the removal treatment than expected simply due to the compositional nature of the data; post hoc Tukey *p* value ≤ 0.05; Supplementary Table 1). Among the prokaryotic OTUs with an increased relative abundance, 28.2%, 15.5%, and 13.9% were associated with α-Proteobacteria, γ-Proteobacteria, and Bacilli, respectively (Supplementary Figure 5a). Among the eukaryotic OTUs with an increased relative abundance, 56.6% belonged to the Ascomycota class, which were mainly stimulated in the pH2 treatment (Supplementary Figure 5b).

### Influence of coalescence on interactions and community diversity

**Fig. 3:**
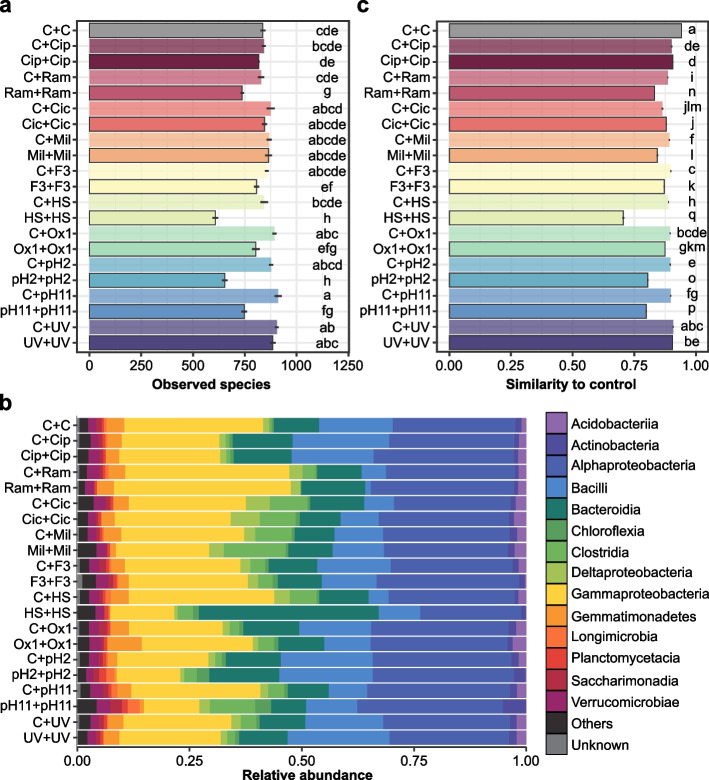
Structure and composition of the prokaryotic communities after Step 2. **a** Number of observed species (mean ± s.e.). The letters indicate significantly different statistical groups (Tukey’s test, *p* value ≤ 0.05). **b** Relative abundances of the fourteen most abundant class of prokaryotic community. **c** Similarity between the control samples and between the control and either the self-mixed removal treatment or the coalescence treatment, based on the Weighted UniFrac distances (mean ± s.e.). The letters indicate significantly different statistical groups (Adonis pairwise comparison, Benjamini-Hochberg corrected *p* value ≤ 0.05)

In Step 2, the soils from 10 removal treatments were mixed into new sterile soil microcosms either by themselves (self-mixed removal treatment, R+R) or with the soil from the Step 1 control (coalescence treatment, R+C). The soil from the Step 1 control was also mixed with itself (C+C) as a new control for this Step 2 (Fig. 1). We consider that coalescence led (i) to full recovery when no significant difference was observed between the coalescence treatment and the Step 2 control and (ii) to partial recovery when a significant difference was observed between the coalescence treatment and the self-mixed removal treatment as well as between the coalescence treatment and the Step 2 control. In 72% of the cases, a higher *α*-diversity was observed in the coalescence treatments compared to the impacted self-mixed removal treatments including for the communities which were the most impacted by the removal step (post hoc Tukey *p* value ≤ 0.05; Fig. 3a, Supplementary Figures 6a and 7). Mixing the depleted and control communities also resulted in 75% of the coalesced communities being more similar to the self-mixed control than their corresponding self-mixed removal treatments (Adonis pairwise comparison, Benjamini-Hochberg corrected *p* value ≤ 0.05; Fig. 3b, c and Supplementary Figures 6b, c and 8).

To test if coalescence restored the relative abundance of the affected OTUs, we used the generalized linear mixed model. Among the OTUs previously affected by the Step 1 removal treatments (R vs C, where vs means > or <), we distinguished those still exhibiting the same differences between the self-mixed removal treatment and the self-mixed control (R vs C ∩ R+R vs C+C, where ∩ means intersection) from the others (R vs C ∉ R+R vs C+C, where ∉ means excluding). Among the latter, we found that 121 prokaryotic and 58 eukaryotic OTUs showed a significant increased relative abundance in the removal treatment from Step 1 but not in the self-mixed removal treatment from Step 2, compared to their respective controls (R>C ∉ R+R>C+C; post hoc Tukey *p* value ≤ 0.05; Supplementary Table 1). Alongside, 107 prokaryotic and 31 eukaryotic OTUs showed a decreased relative abundance in the removal treatment from Step 1 but not in the self-mixed removal treatment from Step 2 (R<C ∉ R+R<C+C; post hoc Tukey *p* value ≤ 0.05; Supplementary Table 1).

**Fig. 4:**
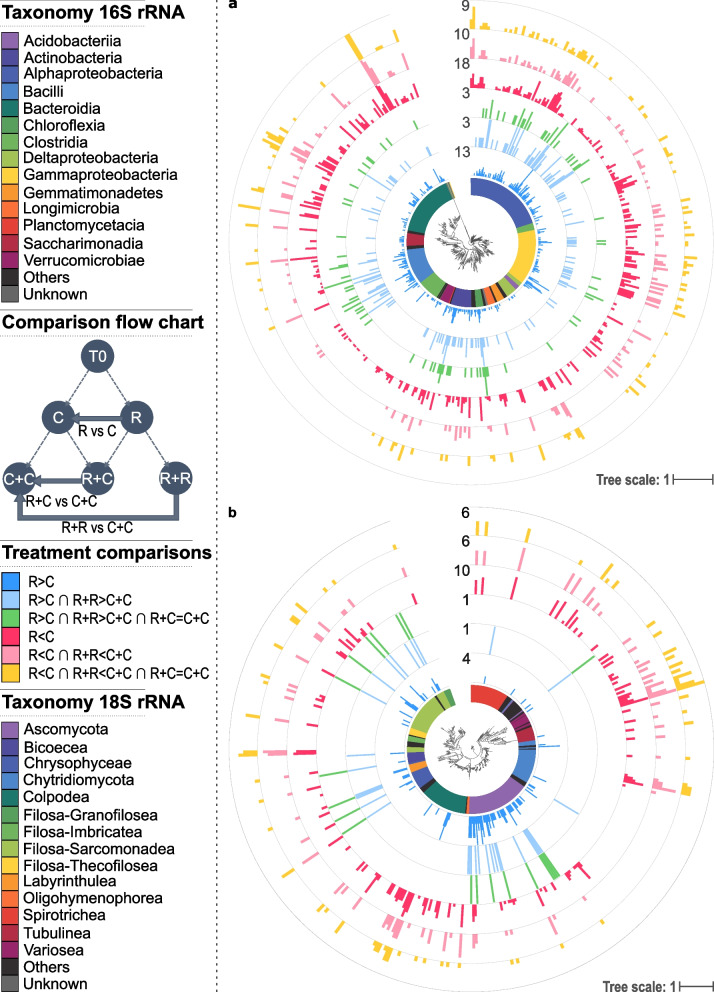
Taxonomic assignment and distribution of significantly differentially abundant OTUs across treatments. Outer rings show prokaryotic (**a**) and eukaryotic (**b**) OTUs significantly affected by the removal treatments (R vs C, where vs means > or <), OTUs significantly affected by both the removal treatments and the self-mixed removal treatments (R vs C ∩ R+R vs C+C, where ∩ means intersection) and OTUs recovering in the coalescence treatments (R vs C ∩ R+R vs C+C ∩ R+C=C+C). Bar scale is proportional to the number of treatment where the OTU is significantly differentially abundant, with the maximum indicated for each ring. The OTU class is indicated by different colors on the innermost ring

Conversely, we found 124 prokaryotic OTUs exhibiting a higher relative abundance in both the removal and the self-mixed removal treatments compared to their respective controls (R>C ∩ R+R>C+C), and among them, 79 no longer showed a significant difference in relative abundance after the coalescence treatments (R>C ∩ R+R>C+C ∩ R+C=C+C; post hoc Tukey *p* value ≤ 0.05; Fig. 4a, Supplementary Table 1, Supplementary Figures 9a, and 10a). These OTUs mostly belong to α-Proteobacteria, Bacilli, and Actinobacteria (Fig. 4a). Fewer eukaryote OTUs exhibited the same affected pattern in both steps (i.e., 32 OTUs R>C ∩ R+R>C+C) and 20 of them no longer showed any significant difference in relative abundance after the coalescence treatments (R>C ∩ R+R>C+C ∩ R+C=C+C; post hoc Tukey *p* value ≤ 0.05; Fig. 4b, Supplementary Table 1, Supplementary Figures 9b, and 10b). Similarly, among the 133 prokaryotic and 72 eukaryotic OTUs that were depleted in removal treatments from both steps (R<C ∩ R+R<C+C), 113 and 61 completely recovered in the coalescence treatments, respectively (R<C ∩ R+R<C+C ∩ R+C=C+C; post hoc Tukey *p* value ≤ 0.05; Fig. 4, Supplementary Table 1, Supplementary Figures 9 and 10). Therefore, among the OTUs that were affected by the removal treatment in both steps, a total 176 prokaryotic and 80 and eukaryotic OTUs fully recovered in the coalescence treatments (R vs C ∩ R+R vs C+C ∩ R+C=C+C; post hoc Tukey *p* value ≤ 0.05; Supplementary Table 1).

Interestingly, an unexpected pattern emerged from coalescence as the relative abundances of 73 prokaryotic and 48 eukaryotic OTUs were significantly higher or lower in at least one of the coalescence treatments compared to each self-mixed source community separately (R+C>C+C ∩ R+C>R+R or R+C<C+C ∩ R+C<R+R, respectively; post hoc Tukey *p* value ≤ 0.05; Supplementary Table 1, Supplementary Figures 11 and 12). These OTUs mostly belong to the bacterial classes γ-Proteobacteria and Bacteroidia and to the protist classes Filosa-Sarcomonadea and Colpodea (Supplementary Figure 12).

### Network inference deciphers biotic interactions

**Fig. 5:**
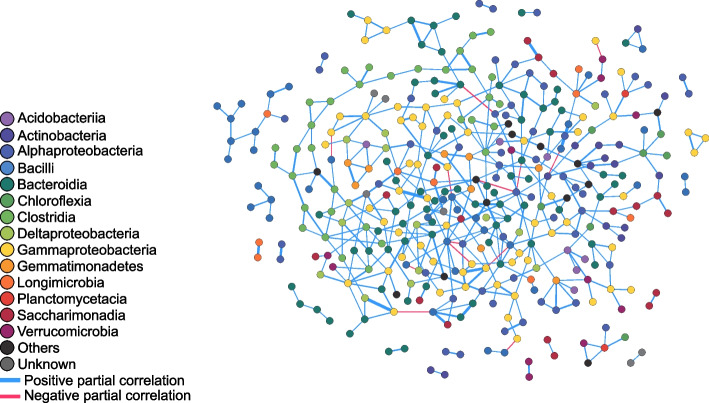
Global prokaryotic network inferred from all samples across both experimental steps. Nodes represent OTUs and they are colored according to the OTU taxonomic class. Links represent partial correlations *ρ*, and they are colored blue if *ρ* > 0 and red if *ρ* < 0. Link width is proportional to |*ρ*|

To identify antagonistic OTUs, we inferred co-occurrence networks across all samples using a sparse multivariate Poisson log-normal model [42]. Among the significant negative links inferred in the prokaryotic network, 320 out of 1383 were connecting Proteobacteria with either Bacteroidia or Bacilli (Supplementary Figure 13). However, the strength of the links was the highest for those connecting γ-Proteobacteria and Bacilli, which represented up to 46.2% of the strongest negative links with a partial correlation threshold of |*ρ*| > 0.08 (Fig. 5 and Supplementary Figure 13). The number of negative links was much lower for the eukaryotic network with 110 links and only two between four protist OTUs with a partial correlation threshold |*ρ*| > 0.08 (Supplementary Figure 14). Only one positive partial-correlation between a γ-Proteobacteria and a Chrysophyceae was observed across domains (partial correlation |*ρ*| > 0.08; Supplementary Figure 15).

### Responses of soil properties and functions to microbial community manipulations

**Fig. 6:**
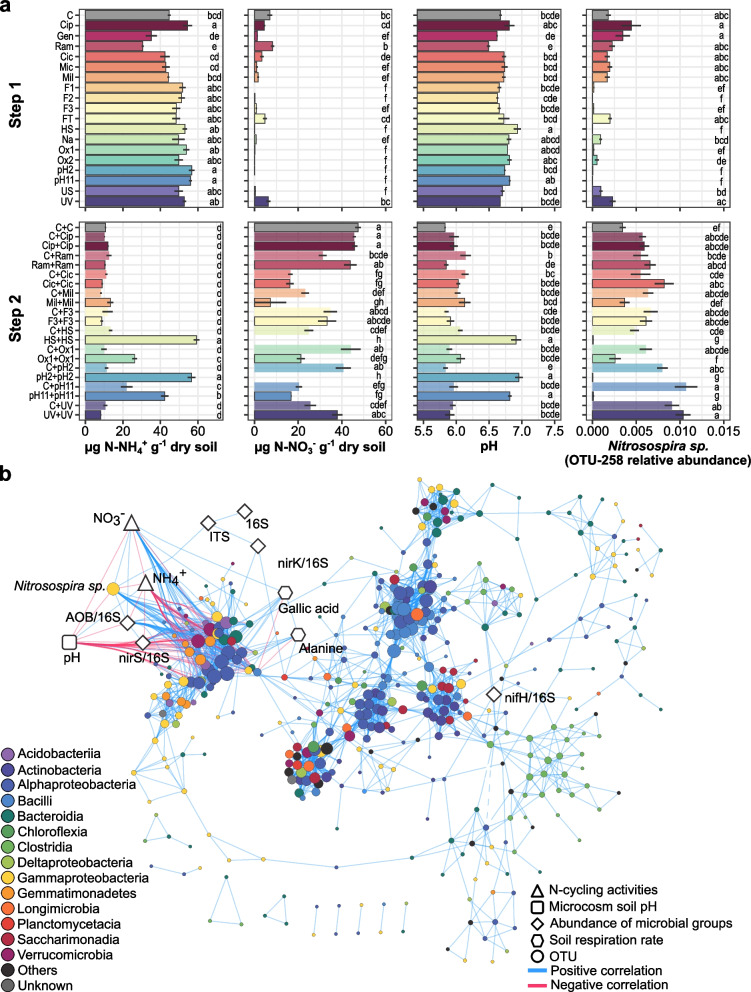
Treatment-induced changes in soil properties and N-cycling microbial guilds and inferred correlations with microbial OTUs. **a** Treatment-induced changes in soil inorganic nitrogen pools (NH4+ and NO3-), soil pH, and relative abundance of the OTU-258 (*Nitrosospira sp.*) in the removal treatments and the control (Step 1) as well as in the self-mixed removal treatment, the coalescence treatment, and the self-mixed control (Step 2). The letters indicate significantly different statistical groups (Tukey’s test, *p* value ≤ 0.05). **b** Data integration analysis of 16S rRNA sequences (OTU, circle shaped nodes), inorganic N pools (triangle-shaped nodes), soil pH (square-shaped node), abundances of N-cycling microbial guilds (diamond-shaped nodes), and soil respiration rates (hexagon-shaped nodes) in all samples, regardless the step or treatment. The taxonomic identities of the OTUs are indicated at the class level. The links indicate positive (blue) or negative (red) correlation (Pearson’s correlation |*r*|> 0.6)

We determined treatment-induced changes in soil properties and functions by measuring soil pH, soil respiration rate as proxies of C-cycling, inorganic nitrogen pools and abundance of ammonia-oxidizers, denitrifiers, and diazotrophs as proxies of N-cycling (Fig. 6, Supplementary Figures 16 and 17). The depletion treatments did not significantly affect community respiration rates whatever the substrate (post hoc Tukey *p* value ≤ 0.05; Supplementary Figure 16). In contrast, almost all depletion treatments lowered the nitrate content compared to the control and 8 out of 18 treatments resulted in a significant decrease in the abundance of ammonia-oxidizing bacteria (AOB; post hoc Tukey *p* value ≤ 0.05; Supplementary Figure 17a). Only the pH2 and heat-shock treatments affected the abundance of all the studied N-cycling communities (post hoc Tukey *p* value ≤ 0.05; Supplementary Figure 17a, b, c, d). While only the heat shock treatment affected the soil pH in Step 1, three self-mixed removal treatments (HS+HS, pH2+pH2, pH11+pH11) had a significantly much higher pH than other treatments (post hoc Tukey *p* value ≤ 0.05; Fig. 6a). These same three self-mixed treatments also displayed a higher ammonium content as well as lower nitrate content and AOB abundances than the control (post hoc Tukey *p* value ≤ 0.05; Fig. 6a and Supplementary Figure 17e). Meanwhile, the three corresponding coalescence treatments (C+HS, C+pH2, C+pH11) displayed partly or fully restored pH, ammonium pool, and AOB community abundance compared to the Step 2 control (post hoc Tukey *p* value ≤ 0.05; Fig. 6a and Supplementary Figure 17e). To statistically infer correlations between these proxies and microbial OTUs, we used a multi-omics integrative approach based on Projection to Latent Structure [46] (Pearson’s correlation |*r*|> 0.6; Fig. 6b). We found that pH was negatively correlated with the nitrate concentration as well as the AOB to 16S ratio while being positively correlated with the ammonium concentration. The latter was itself negatively correlated with the nitrate concentration and the AOB to 16S ratio. We found that an OTU belonging to a well-known group of ammonia-oxidizers—*Nitrosospira* sp.—was also positively correlated with the AOB to 16S ratio and negatively correlated with the ammonium concentration and the soil pH (Pearson’s correlation |*r*|> 0.6; Fig. 6).

## Discussion

While microbial communities are recognized as key drivers of several ecosystem functions, a clear understanding of the factors shaping their assembly is still missing. The influence of soil abiotic properties on microorganisms has been reported in a large body of literature. However, the importance of biotic interactions between microorganisms has been overlooked and is still unclear [27, 47]. To assess to which extent microbe-microbe interactions can contribute to microbiome assembly and processes, we combined targeted removal and coalescence approaches to manipulate soil microbial communities [18].

Our removal manipulation experiment showed that about 47.6 % of the dominant prokaryotic OTUs exhibited a significant relative fitness benefit after the depletion treatments. This result is consistent with our previous findings based on another community from a soil with contrasted physico-chemical properties [27]. The number of 18S OTUs showing differential relative abundance between the removal treatments and the control was much lower, which does not necessarily suggest that biotic interactions involving eukaryotes are rarer. Rather, our experimental approach might not be suitable for eukaryotic communities since inoculation of the sterile soil resulted in a high variability in the distribution of 18S sequences and in the loss of more than half of the 18S diversity compared to the original soil. This is likely due to intrinsic properties of these communities, and therefore, we will here mostly focus on prokaryotic communities.

To establish a causative relationship between the depletion of some taxa and the increased relative abundance of others observed during Step 1, we performed a targeted coalescence experiment to reunite potentially interacting OTUs. We hypothesized that the OTUs with increased relative abundance after the removal treatment in Step 1 would be detrimentally affected during soil colonization only when mixed with the control community that still contains the OTUs exerting antagonistic interactions (R>C ∩ R+R>C+C ∩ R+C=C+C). To alleviate environmental filtering and priority effects that can also influence the outcome of coalescence [14], the depleted and control communities were mixed into a larger volume of the same sterilized soil (Fig. 1). The integration of the results of the differential abundance analysis of both steps revealed that 79 prokaryotic OTUs supported our hypothesis with coalescence resulting in the complete loss of the relative fitness benefit observed after the removal experiment (post hoc Tukey *p* value ≤ 0.05; Fig. 4a and Supplementary Table 1). This contrasts with a scenario of no interactions under which the OTUs with increased relative abundance after the removal treatment would still exhibit a relative abundance in the coalesced treatment that is different from the control treatment. This indicates that these putative interactions were validated across both steps, which suggests that 15% (79/515 OTUs) of the dominant bacterial taxa were engaged in negative interactions during soil recolonization. Such coalescence approach has previously been successfully used to validate functional interactions within bacterial communities from rainwater pools [48].

We also found that even without coalescence events, several OTUs with increased relative abundance after the removal treatments in Step 1 (R>C) no longer showed significant increased relative abundance in Step 2 (R>C ∉ R+R>C+C; Supplementary Table 1). This can be explained by the re-growth of depleted antagonists in removal treatments during the Step 2, which is supported by the relative abundance of 107 OTUs that were no longer depleted in the self-mixed removal treatments while they were in the Step 1 (R<C ∉ R+R<C+C; Supplementary Table 1). Alternatively, these variations in relative abundances between Step 1 and 2 without coalescence events could also be due to changes in soil abiotic properties induced by differences in microbial community composition during Step 1 and by the mixing of soils from Step 1 with sterile soil in Step 2. These OTUs were therefore discarded when estimating the importance of the interactions between microorganisms since both abiotic and biotic effects could explain this pattern. Nevertheless, when considering only the 229 prokaryotic and 99 eukaryotic OTUs that still exhibited an affected relative abundance in the self-mix removal treatments compared to the self-mixed control (R>C ∩ R+R>C+C and R<C ∩ R+R<C+C), we found that 76.9% and 80.8% of them, respectively, no longer exhibit a difference in relative abundance between the coalescence treatments and the self-mixed control ( R>C ∩ R+R>C+C ∩ R+C=C+C and R<C ∩ R+R<C+C ∩ R+C=C+C ; Fig. 4, Supplementary Table 1). This suggests that the coalescence treatments allowed re-establishing the original interactions for a large majority of the OTUs affected by the depletion treatments in both steps.

The coalescence experiment alone also revealed emergent interactions that could not be predicted from the source communities (Supplementary Table 1 and Supplementary Figure 12). Thus, we identified a total of 73 prokaryotic and 48 eukaryotic OTUs that exhibited significantly higher or lower relative abundances when mixing communities from the control and removal treatments (i.e., R+C) than when mixing each source community separately (i.e., C+C and R+R). Since all species are present in the control community, these non-additive changes in relative abundances are likely not due to new higher-order interactions, which could have occurred only if additional species would have been introduced [49, 50]. This pattern, however, could be explained by differences in the relative abundances of interacting OTUs between the source communities. Consistent with this view, density dependence was reported as a key feature characterizing interspecific interactions [51] and pairwise competition experiments demonstrated that species interactions can be influenced by the initial microbial species abundances [11]. Our results based on complex communities complement and extend these previous findings by suggesting that not only such density-dependent interactions may affect the outcome of coalescence but could also account for an important fraction of the observed interactions. About 83.6 and 54.2 % of these non-additive changes in the prokaryotic and eukaryotic communities, respectively, resulted in an increase rather than a decrease of the relative abundance in the coalesced communities compared to the self-mixed source communities, which further suggests that cooperation between microorganisms might not be as rare as previously reported [10].

Next, we used co-occurrence network inference to identify which OTUs were interacting across the different treatments. While Russel et al. [9] reported that antagonism was most likely among closely related species, we found that almost 50% of the negative links in the prokaryotic network were between members of the Proteobacteria and Bacillales. However, the two sets of data are not necessarily contradictory since 5500 prokaryotic OTUs belonging to more than 300 families that were coexisting in the same soil were used here for network inference, while Russel et al. used 65 strains from 8 distinct environments such as soil, freshwater, maize leaf and marine algae. Our findings indicate that some members of the Firmicutes may be outcompeted by γ-Proteobacteria are of importance for understanding community assembly rules in soil. Consistent with our results, Romdhane et al. [27] showed that the relative fitness of Firmicutes benefited from a decrease in γ-Proteobacteria. Interestingly, this assembly rule might hold true in other environments. For example, in vitro and in vivo pairwise competition assays between phyllosphere strains revealed directional antibiosis with Firmicutes being strongly inhibited and outcompeted by a subset of Proteobacteria [52]. However, our experimental approach does not explain the nature of the observed antagonistic interactions and therefore additional work would be needed to identify the underlying mechanisms. Nevertheless, the link between γ-Proteobacteria and Chrysophyceae, which was the only one observed across domains, is consistent with previous findings reporting that members of the latter could be mixotrophic bacterivores feeding on Proteobacteria [53].

Until recently, soil microbes have seldom been considered as important players for ecological restoration of degraded ecosystems [16]. Our coalescence approach consisting in mixing the depleted and control microbial communities resulted in asymmetrical outcome with the coalesced communities being more similar to the control community from the Step 2 than to the depleted ones. Diversity of both eukaryotic and prokaryotic communities as well as their functions were partly or fully restored after coalescence even in some of the most impaired treatments. In line with our results, previous studies showed that communities that are more efficient at using resources will dominate in coalescence events [54–56]. Wubs et al. [17] recently reported that soil inocula could steer plant communities and promote ecosystem restoration in the field. In contrast, previous studies using inoculation of microbial communities often failed to prove consistent effectiveness, which was attributed to unfavorable biotic or abiotic conditions in the receptor soils [57–59]. Here, the integration of the different data sets in a supervised analysis (Fig. 6) revealed that changes in the ammonium pools were due to impaired nitrification, which was partly or fully restored after increasing the AOB in the coalesced communities. That slow-growing nitrifying bacteria were not outcompeted during coalescence and range expansion for recolonization of the sterile soil shows promise in the possibility to steer even fastidious microorganisms for the recovery of degraded ecosystems. Although in our work impaired nitrification was due to the depletion of nitrifiers through artificial manipulation of the soil microbiome, recent work showed that N- and C-cycling in natural ecosystems such as permafrost soils could also be limited by the absence or the low abundance of the corresponding microbial guilds [60]. Another interesting feature emerging from this analysis is that manipulation of microbial community composition can lead to changes in soil pH only within a few weeks. This brings a new dimension to studies investigating the relationships between soil properties and microbiome composition. Thus, soil pH is mostly considered as a major driver of soil microbial communities [61] while the opposite has seldom been addressed [62].

## Conclusions

In conclusion, our top-down approach combining removal and coalescence manipulation of soil microbial communities not only enabled exploration of interactions between soil microorganisms but also allowed linking community structure and ecosystem functions. Our data also highlight the importance of density-dependent interactions for soil bacterial community assembly. Coalescence between manipulated and non-manipulated communities re-established the original interactions and restored—at least partly—both microbial community diversity and functions, which open up new perspectives to steer microbial communities for ecosystem restoration. Finally, our findings that shifts in microbial community composition can lead to significant changes in soil pH warrant further studies to determine the importance of the linkages as well as of the feedback effects between soil biotic and abiotic properties.

## Supplementary Information

**Additional file 1: Supplementary Table 1.** Number of OTUs significantly differentially abundant among all treatments as estimated using a generalized linear mixed model, among the 515 most abundant 16S rRNA OTUs and the 439 most abundant 18S rRNA OTUs. **Supplementary Figure 1.** Abundances of total bacteria and total fungi. Quantification of 16S rRNA (a and c) and ITS (b and d) gene copy numbers in the original soil, the removal treatments and the control (Step 1; a and b) and in the coalescence treatment, the self-mixed removal treatment and the control samples (Step 2; c and d) (mean ± s.e. of log10-transformed data expressed as gene copy g-1 dry soil). Letters indicate significantly different statistical groups (Tukey’s test, p-value ≤ 0.05). **Supplementary Figure 2.** Diversity of prokaryotic and eukaryotic communities after Step 1. The Faith’s phylogenetic diversity (a and c) and Simpson’s reciprocal (b and d) indices are shown (mean ± s.e.) in the original soil, the removal treatments and the control (Step 1) for the 16S rRNA OTUs (a and b) and the 18S rRNA OTUs (c and d). Letters indicate significantly different statistical groups (Tukey’s test, p-value ≤ 0.05). **Supplementary Figure 3.** Structure and composition of the eukaryotic communities in the original soil and after Step 1. (a) Number of observed species (mean ± s.e.). Letters indicate significantly different statistical groups (Tukey’s test, p-value ≤ 0.05). (b) Relative abundances of the fourteen most abundant classes of eukaryotic community. (c) Similarity between the control samples and between the control and either the original soil or the removal treatments, based on the Weighted UniFrac distances (mean ± s.e.). Letters indicate significantly different statistical groups (Adonis pairwise comparison, Benjamini-Hochberg corrected p-value ≤ 0.05). **Supplementary Figure 4.** Principal coordinate analysis (PCoA) of the prokaryotic (a) and eukaryotic (b) communities, based on the weighted UniFrac distance matrix showing the original soil, the removal treatment and the control samples and the 95% joint confidence ellipse for the control samples. **Supplementary Figure 5.** Taxonomic relationships and distribution of OTUs significantly affected by the Step 1 removal treatments compared to the Step 1 control. The outer rings show the effect of each removal treatment on the relative abundances of the 515 most abundant 16S rRNA OTUs (a) and the 439 most abundant 18S rRNA OTUs (b) compared to the control (R vs C), as estimated using a generalized linear mixed model. The blue and red boxes in the outer rings indicate OTUs with increasing and decreasing fitness respectively, while white boxes indicate OTUs that are not affected by the treatment. The OTU class level is indicated by different colors on the innermost ring. **Supplementary Figure 6.** Structure and composition of the eukaryotic communities after Step 2. (a) Number of observed species (mean ± s.e.). Letters indicate significantly different statistical groups (Tukey’s test, p-value ≤ 0.05). (b) Relative abundances of the fourteen most abundant class of eukaryotic community. (c) Similarity between the control samples and between the control and either the self-mixed removal treatment or the coalescence treatments based on the Weighted UniFrac distances (mean ± s.e.). Letters indicate significantly different statistical groups (Adonis pairwise comparison, Benjamini-Hochberg corrected p-value ≤ 0.05). **Supplementary Figure 7.** Diversity levels of prokaryotic and eukaryotic communities after Step 2. The Faith’s phylogenetic diversity (a and b) and Simpson’s reciprocal (c and d) indices are shown (mean ± s.e.) in the coalescence treatment, the self-mixed removal treatments and the control samples (Step 2) for the 16S rRNA OTUs (a and c) and the 18S rRNA OTUs (b and d). Letters indicate significantly different statistical groups (Tukey’s test, p-value ≤ 0.05). **Supplementary Figure 8.** Principal coordinate analysis (PCoA) of the prokaryotic (a) and eukaryotic (b) communities, based on the weighted UniFrac distance matrix showing the self-mixed removal treatment, the coalescence treatment and the control samples and the 95% joint confidence ellipse for the control samples. **Supplementary Figure 9.** Taxonomic relationships and distribution of OTUs significantly affected by the Step 2 removal treatments compared to the Step 2 control. The outer rings show the effect of each self-mixed removal treatment on the relative abundance of the 515 most abundant 16S rRNA OTUs (a) and the 439 most abundant 18S rRNA OTUs (b) compared to the self-mixed control (R+R vs C+C), as estimated using a generalized linear mixed model. The blue and red boxes in the outer rings indicate OTUs with increasing and decreasing fitness respectively, while white boxes indicate OTUs that are not affected by the treatment. The OTU class level is indicated by different colors on the innermost ring. **Supplementary Figure 10.** Taxonomic relationships and distribution of OTUs significantly affected by the Step 2 coalescence treatments compared to the Step 2 control. The outer rings show the effect of each coalescence treatment on the relative abundance of the 515 most abundant 16S rRNA OTUs (a) and the 439 most abundant 18S rRNA OTUs (b) compared to the self-mixed control (R+C vs C+C), as estimated using a generalized linear mixed model. The blue and red boxes in the outer rings indicate OTUs with increasing and decreasing fitness respectively, while white boxes indicate OTUs that are not affected by the treatment. The OTU class level is indicated by different colors on the innermost ring. **Supplementary Figure 11.** Taxonomic relationships and distribution of OTUs significantly affected by the Step 2 coalescence treatments compared to the Step 2 removal treatments. The outer rings show the effect of each coalescence treatment on the relative abundance of the 515 most abundant 16S rRNA OTUs (a) and the 439 most abundant 18S rRNA OTUs (b) compared to its corresponding self-mixed removal treatment (R+C vs R+R), as estimated using a generalized linear mixed model. The blue and red boxes in the outer rings indicate OTUs with increasing and decreasing fitness respectively, while white boxes indicate OTUs that are not affected by the treatment. The OTU class level is indicated by different colors on the innermost ring. **Supplementary Figure 12.** Taxonomic relationships and distribution of significantly differentially abundant OTUs across treatments. Outer rings show prokaryotic (a) and eukaryotic (b) OTUs exhibiting relative abundances significantly higher or lower in the coalescence treatments than in the self-mixed source community separately (R+C>C+C ∩ R+C>R+R and R+C<C+C ∩ R+C<R+R). Bar scale is proportional to the number of treatment where the OTU is significantly differentially abundant, with a maximum indicated for each comparison ring. The OTU class is indicated by different colors on the innermost ring. **Supplementary Figure 13.** Number of negative links between prokaryotic taxa. Number of links represents significant negative partial correlation from the global prokaryotic network inferred from all samples across both experimental steps. The insert represents the proportion of negative links for the top three taxa depending on the strength of the partial correlations. **Supplementary Figure 14.** Global eukaryotic network inferred from all samples across both experimental steps. Nodes represent OTUs and they are colored according to the OTU taxonomic class. Edges represent partial correlations ρ and they are colored blue if ρ > 0 and red if ρ < 0. Edge width is proportional to |ρ|. **Supplementary Figure 15.** Global inter-domain network inferred from all samples across both experimental steps. Nodes represent OTUs and they are colored according to the OTU taxonomic class. Edges represent partial correlations ρ and they are colored blue if ρ > 0 and red if ρ < 0. Edge width is proportional to |ρ|. **Supplementary Figure 16.** Treatment-induced changes in soil respiration rate as proxies of C-cycling. Substrate-induced respiration was measured by the MicroResp™ method using the substrates alanine (a and d), fructose (b and e) and gallic acid (c and f) in the original soil, the removal treatments and the control (Step 1; a, b and c) or in the coalescence treatments and the self-mixed treatments (Step 2; d, e and f) (mean ± s.e.). Letters indicate significantly different statistical groups (Tukey’s test, p-value ≤ 0.05). **Supplementary Figure 17.** Treatment-induced changes in abundance of N-cycle microbial guilds as proxies of N-cycling. Abundances of ammonia-oxidizing bacteria (AOB in a and e), bacterial denitrifiers (nirK in b and f; nirS in c and g) and nitrogen-fixing bacteria (nifH in d and h) in the original soil, the removal treatments and the control (Step 1; a, b, c and d) or in the coalescence treatments and the self-mixed treatments (Step 2; e, f, g and h) (mean ± s.e. of log10-transformed data expressed as gene copy g-1 dry soil). Letters indicate significantly different statistical groups (Tukey’s test, p-value ≤ 0.05).

## Data Availability

Raw sequences were deposited at the NCBI under the accession number PRJNA763056 for 16S rRNA sequences and PRJNA763098 for the 18S rRNA sequences. All code and data are available on gitlab at the following link: https://gitlab.com/micro_bio_info_sarah/huet_2021.
