# Supplementary material for: Experimental community coalescence sheds light on microbial interactions in soil and restores impaired functions
Source: Microbiome. 2023 Mar 4;11:42. doi: 10.1186/s40168-023-01480-7 (PMC9985222; doi:10.1186/s40168-023-01480-7)
Supplement: Supplementary file 2 — Additional file 1: Supplementary Table 1. Number of OTUs significantly differentially abundant among all treatments as estimated using a generalized linear mixed model, among the 515 most abundant 16S rRNA OTUs and the 439 most abundant 18S rRNA OTUs. Supplementary Figure 1. Abundances of total bacteria and total fungi. Quantification of 16S rRNA (a and c) and ITS (b and d) gene copy numbers in the original soil, the removal treatments and the control (Step 1; a and b) and in the coalescence treatment, the self-mixed removal treatment and the control samples (Step 2; c and d) (mean ± s.e. of log10-transformed data expressed as gene copy g-1 dry soil). Letters indicate significantly different statistical groups (Tukey’s test, p-value ≤ 0.05). Supplementary Figure 2. Diversity of prokaryotic and eukaryotic communities after Step 1. The Faith’s phylogenetic diversity (a and c) and Simpson’s reciprocal (b and d) indices are shown (mean ± s.e.) in the original soil, the removal treatments and the control (Step 1) for the 16S rRNA OTUs (a and b) and the 18S rRNA OTUs (c and d). Letters indicate significantly different statistical groups (Tukey’s test, p-value ≤ 0.05). Supplementary Figure 3. Structure and composition of the eukaryotic communities in the original soil and after Step 1. (a) Number of observed species (mean ± s.e.). Letters indicate significantly different statistical groups (Tukey’s test, p-value ≤ 0.05). (b) Relative abundances of the fourteen most abundant classes of eukaryotic community. (c) Similarity between the control samples and between the control and either the original soil or the removal treatments, based on the Weighted UniFrac distances (mean ± s.e.). Letters indicate significantly different statistical groups (Adonis pairwise comparison, Benjamini-Hochberg corrected p-value ≤ 0.05). Supplementary Figure 4. Principal coordinate analysis (PCoA) of the prokaryotic (a) and eukaryotic (b) communities, based on the weighted UniFrac distance matrix [file 40168_2023_1480_MOESM1_ESM.pdf]

Supplementary informations

Experimental community coalescence sheds light on microbial interactions in soil and restores impaired functions

Sarah Huet[1], Sana Romdhane[1], Marie-Christine Breuil[1], David Bru[1], Arnaud Mounier[1], Ayme Spor[1], Laurent Philippot[1]*.

[1]University Bourgogne Franche-Comte, INRAE, AgroSup Dijon, Department of Agroecology, Dijon, France

*Corresponding author: Laurent Philippot laurent.philippot@inrae.fr

Supplementary Table 1: Number of OTUs significantly differentially abundant among all treatments as estimated using a generalized linear mixed model, among the 515 most abundant 16S rRNA OTUs and the 439 most abundant 18S rRNA OTUs.

Comparisons		16S OTU count		18S OTU count	
**R>C**	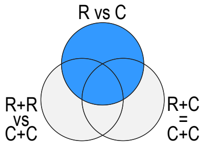	245	375	90	164
**R<C**		240		103	
**R>C ∉ R+R>C+C**	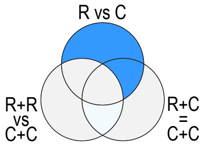	121	228	58	89
**R<C ∉ R+R<C+C**		107		31	
**R>C ∩ R+R>C+C**	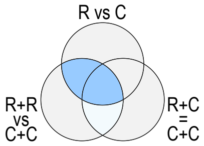	124	229	32	99
**R<C ∩ R+R<C+C**		133		72	
**R>C ∩ R+R>C+C ∩ R+C=C+C**	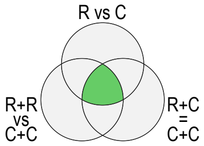	79	176	20	80
**R<C ∩ R+R<C+C ∩ R+C=C+C**		113		61	
**R+C>C+C ∩ R+C>R+R**	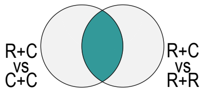	61	73	26	48
**R+C<C+C ∩ R+C<R+R**		16		22	

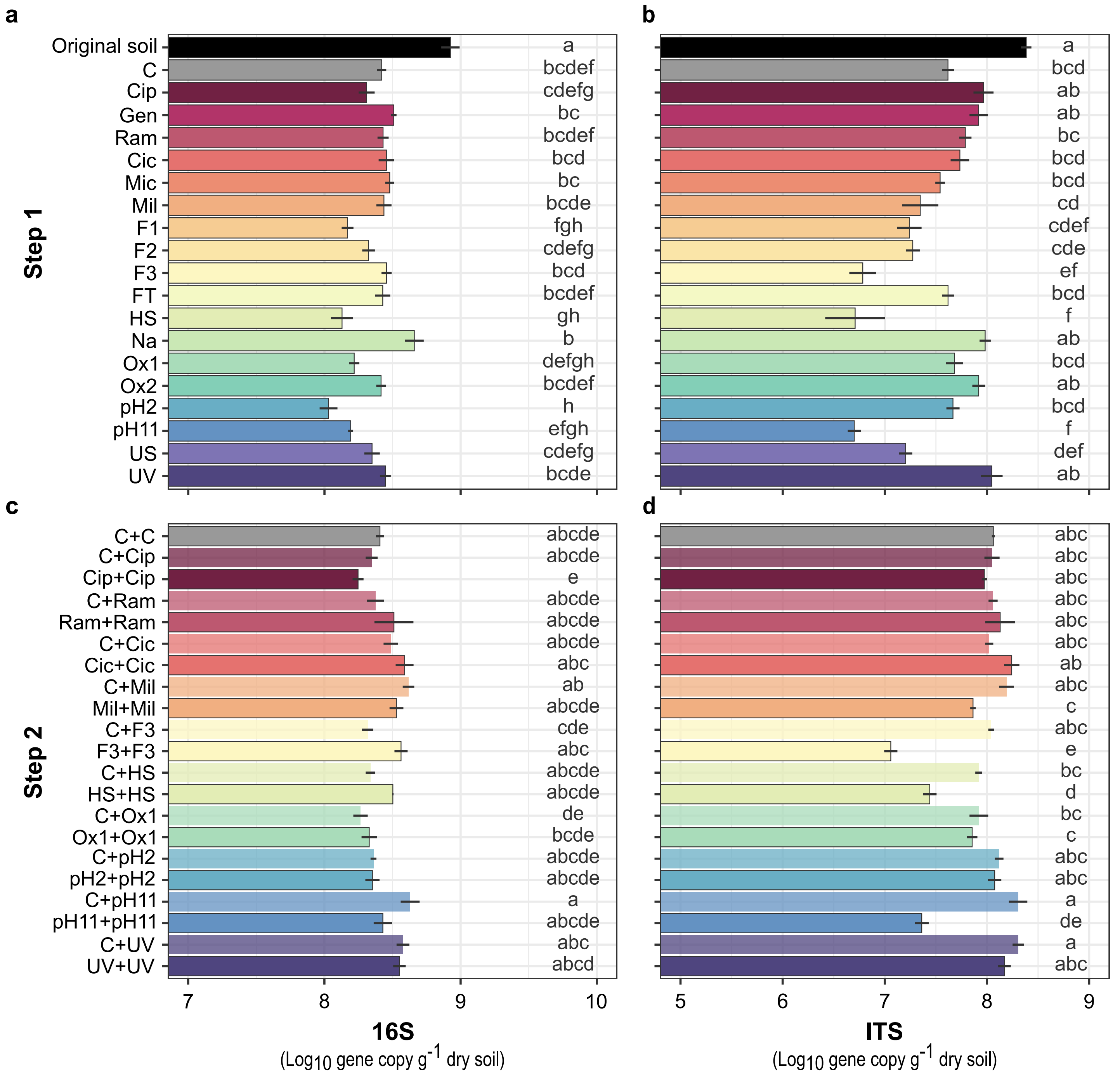

Supplementary Figure 1: Abundances of total bacteria and total fungi. Quantification of 16S rRNA (a and c) and ITS (b and d) gene copy numbers in the original soil, the removal treatments and the control (Step 1; a and b) and in the coalescence treatment, the self-mixed removal treatment and the control samples (Step 2; c and d) (mean ± s.e. of log10-transformed data expressed as gene copy g-1 dry soil). Letters indicate significantly different statistical groups (Tukey's test, p-value ≤ 0.05).

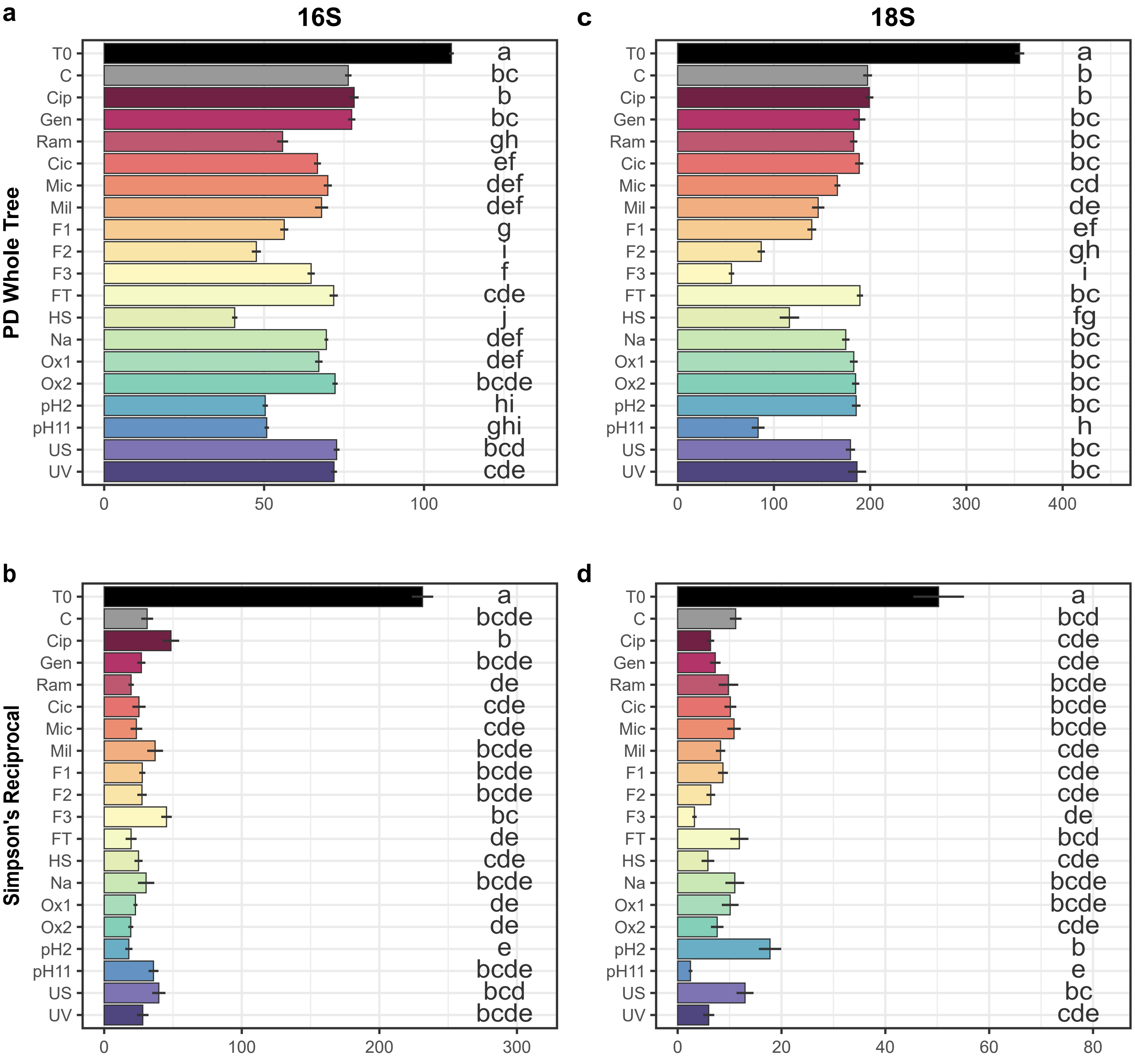

Supplementary Figure 2: Diversity of prokaryotic and eukaryotic communities after Step 1. The Faith's phylogenetic diversity (a and c) and Simpson's reciprocal (b and d) indices are shown (mean ± s.e.) in the original soil, the removal treatments and the control (Step 1) for the 16S rRNA OTUs (a and b) and the 18S rRNA OTUs (c and d). Letters indicate significantly different statistical groups (Tukey's test, p-value ≤ 0.05).

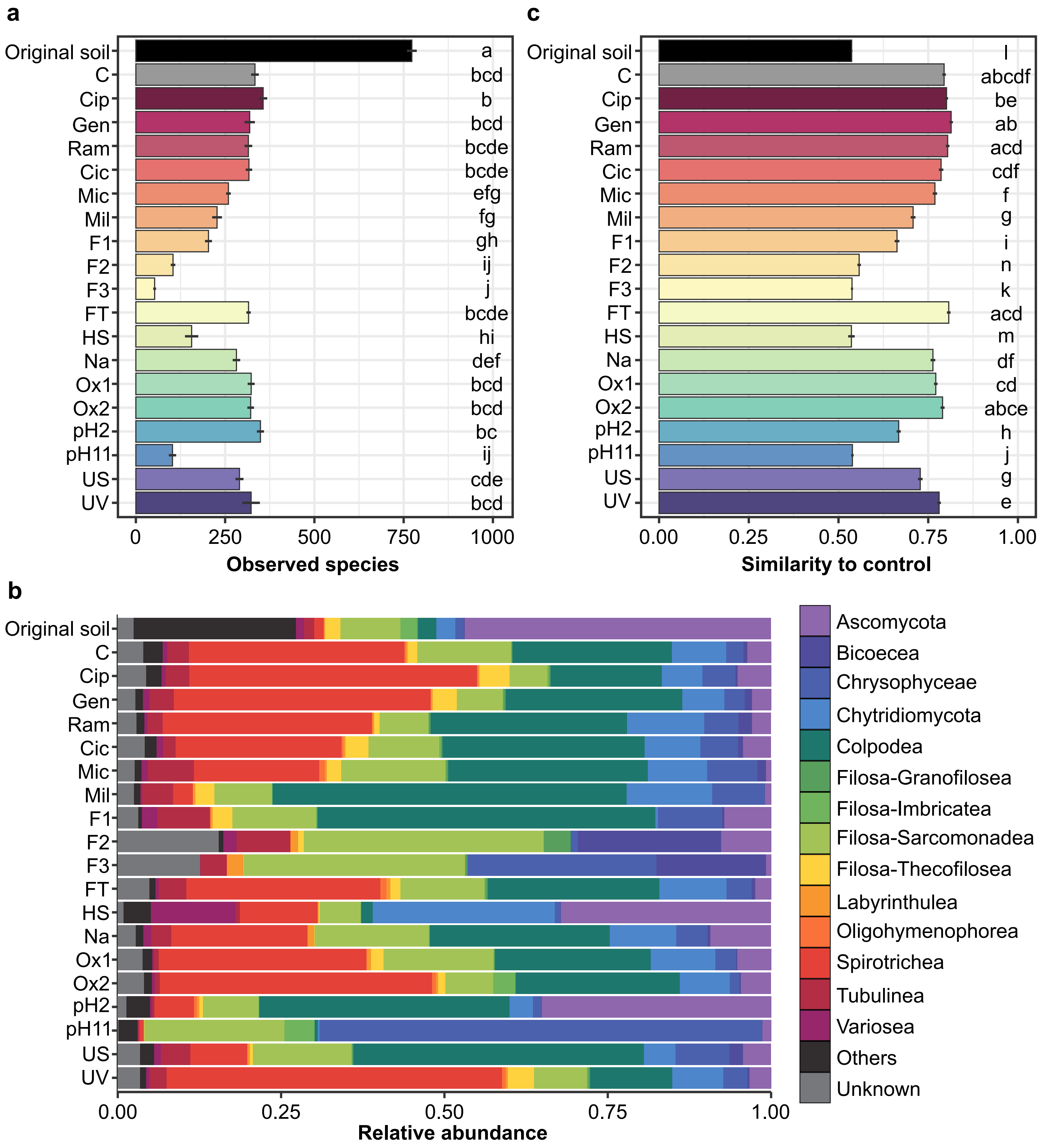

Supplementary Figure 3: Structure and composition of the eukaryotic communities in the original soil and after Step 1. (a) Number of observed species (mean ± s.e.). Letters indicate significantly different statistical groups (Tukey's test, p-value ≤ 0.05). (b) Relative abundances of the fourteen most abundant classes of eukaryotic community. (c) Similarity between the control samples and between the control and either the original soil or the removal treatments, based on the Weighted UniFrac distances (mean ± s.e.). Letters indicate significantly different statistical groups (Adonis pairwise comparison, Benjamini-Hochberg corrected p-value ≤ 0.05).

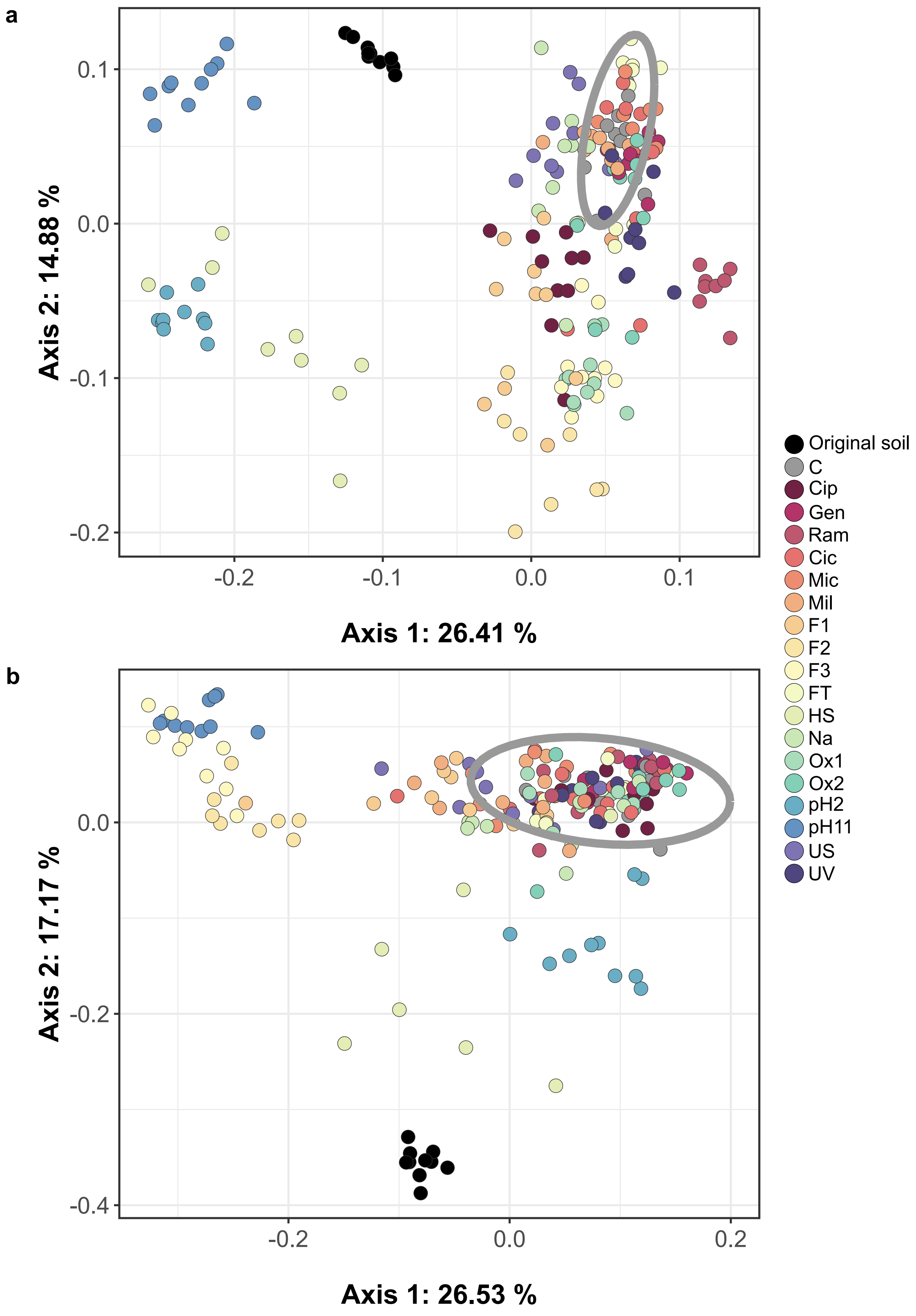

Supplementary Figure 4: Principal coordinate analysis (PCoA) of the prokaryotic (a) and eukaryotic (b) communities, based on the weighted UniFrac distance matrix showing the original soil, the removal treatment and the control samples and the 95% joint confidence ellipse for the control samples.

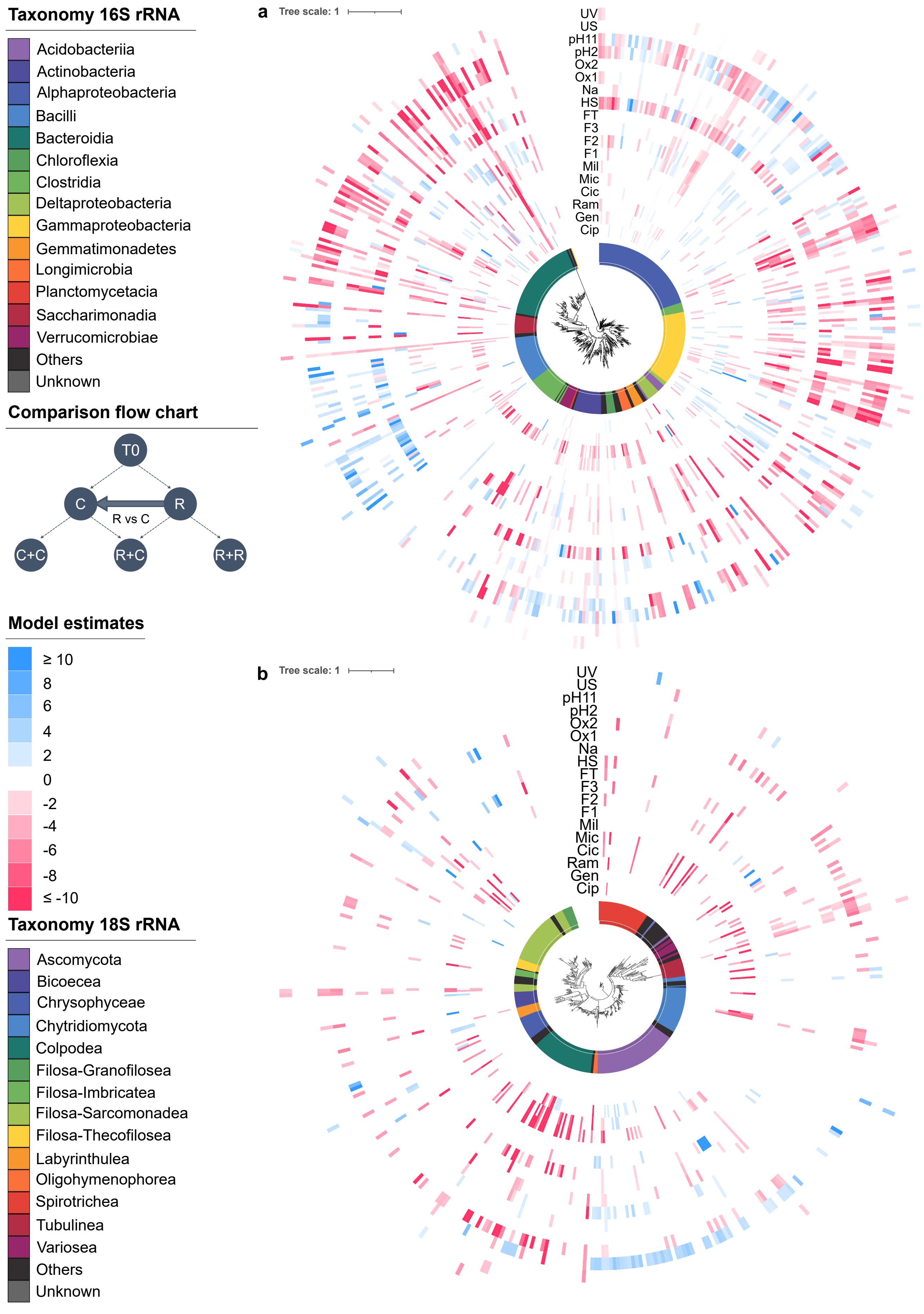

Supplementary Figure 5: Taxonomic relationships and distribution of OTUs significantly affected by the Step 1 removal treatments compared to the Step 1 control. The outer rings show the effect of each removal treatment on the relative abundances of the 515 most abundant 16S rRNA OTUs (a) and the 439 most abundant 18S rRNA OTUs (b) compared to the control (R vs C), as estimated using a generalized linear mixed model. The blue and red boxes in the outer rings indicate OTUs with increasing and decreasing fitness respectively, while white boxes indicate OTUs that are not affected by the treatment. The OTU class level is indicated by different colors on the innermost ring.

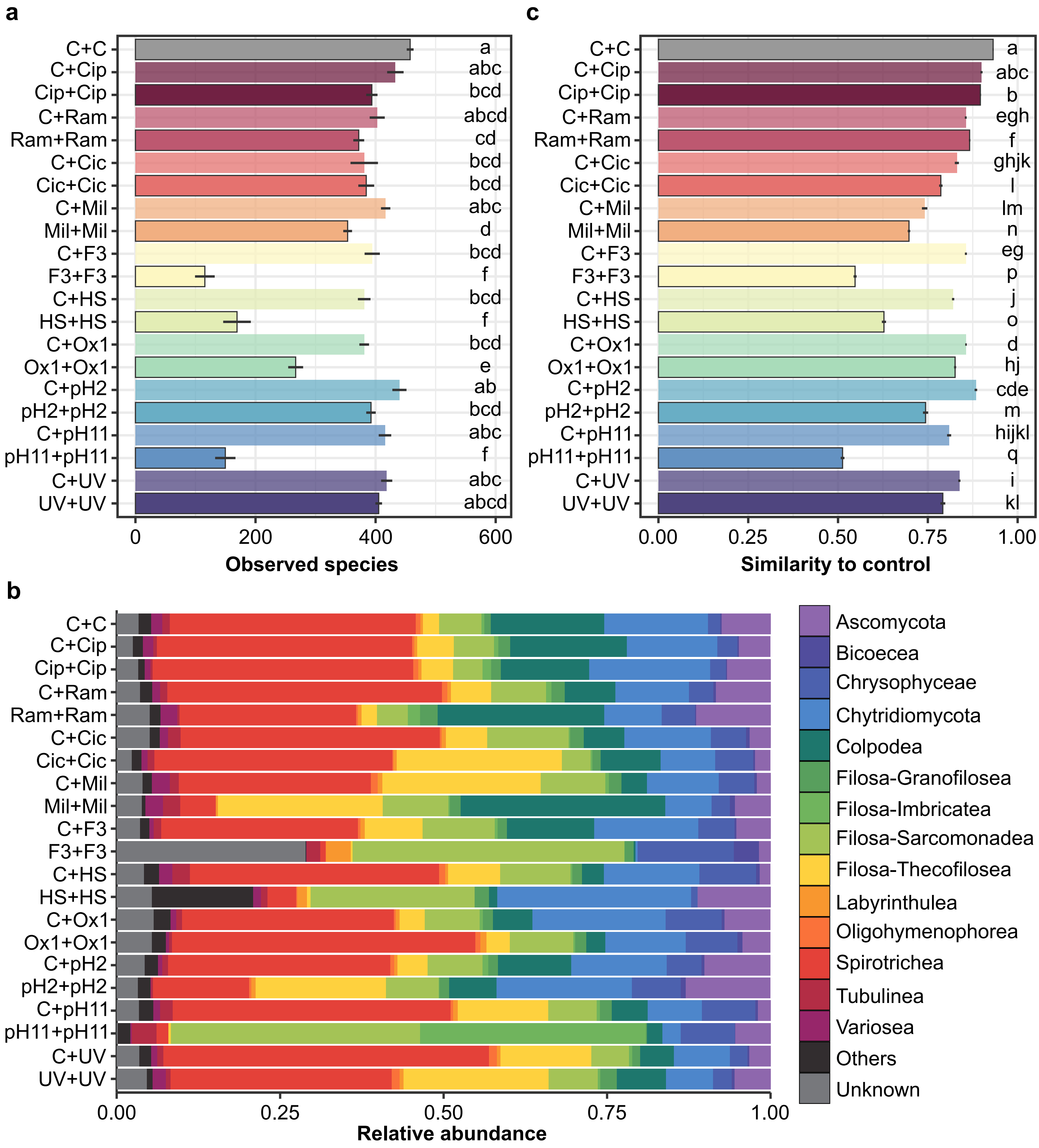

Supplementary Figure 6: Structure and composition of the eukaryotic communities after Step 2. (a) Number of observed species (mean ± s.e.). Letters indicate significantly different statistical groups (Tukey's test, p-value ≤ 0.05). (b) Relative abundances of the fourteen most abundant class of eukaryotic community. (c) Similarity between the control samples and between the control and either the self-mixed removal treatment or the coalescence treatments based on the Weighted UniFrac distances (mean ± s.e.). Letters indicate significantly different statistical groups (Adonis pairwise comparison, Benjamini-Hochberg corrected p-value ≤ 0.05).

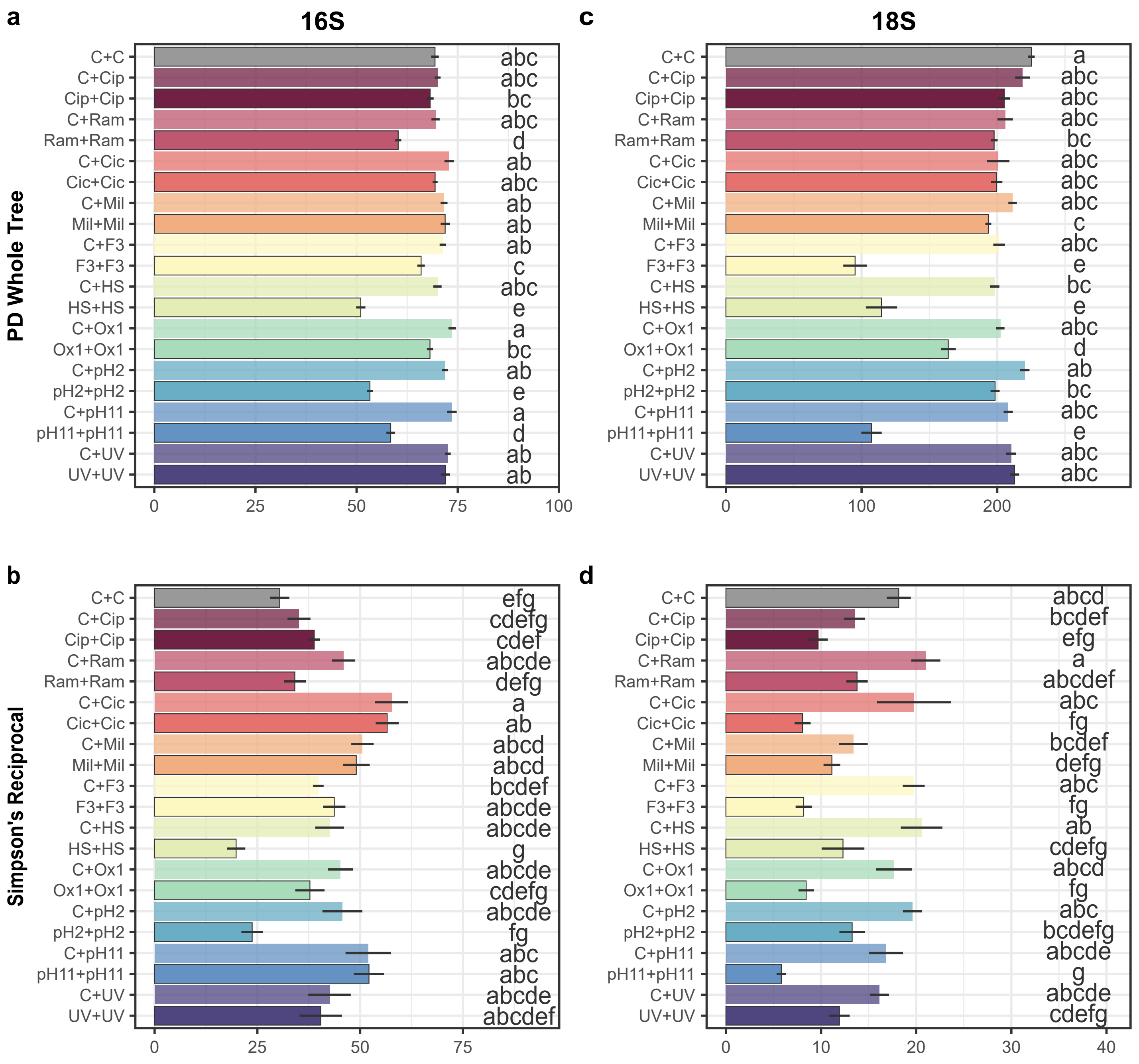

Supplementary Figure 7: Diversity levels of prokaryotic and eukaryotic communities after Step 2. The Faith's phylogenetic diversity (a and b) and Simpson's reciprocal (c and d) indices are shown (mean ± s.e.) in the coalescence treatment, the self-mixed removal treatments and the control samples (Step 2) for the 16S rRNA OTUs (a and c) and the 18S rRNA OTUs (b and d). Letters indicate significantly different statistical groups (Tukey's test, p-value ≤ 0.05).

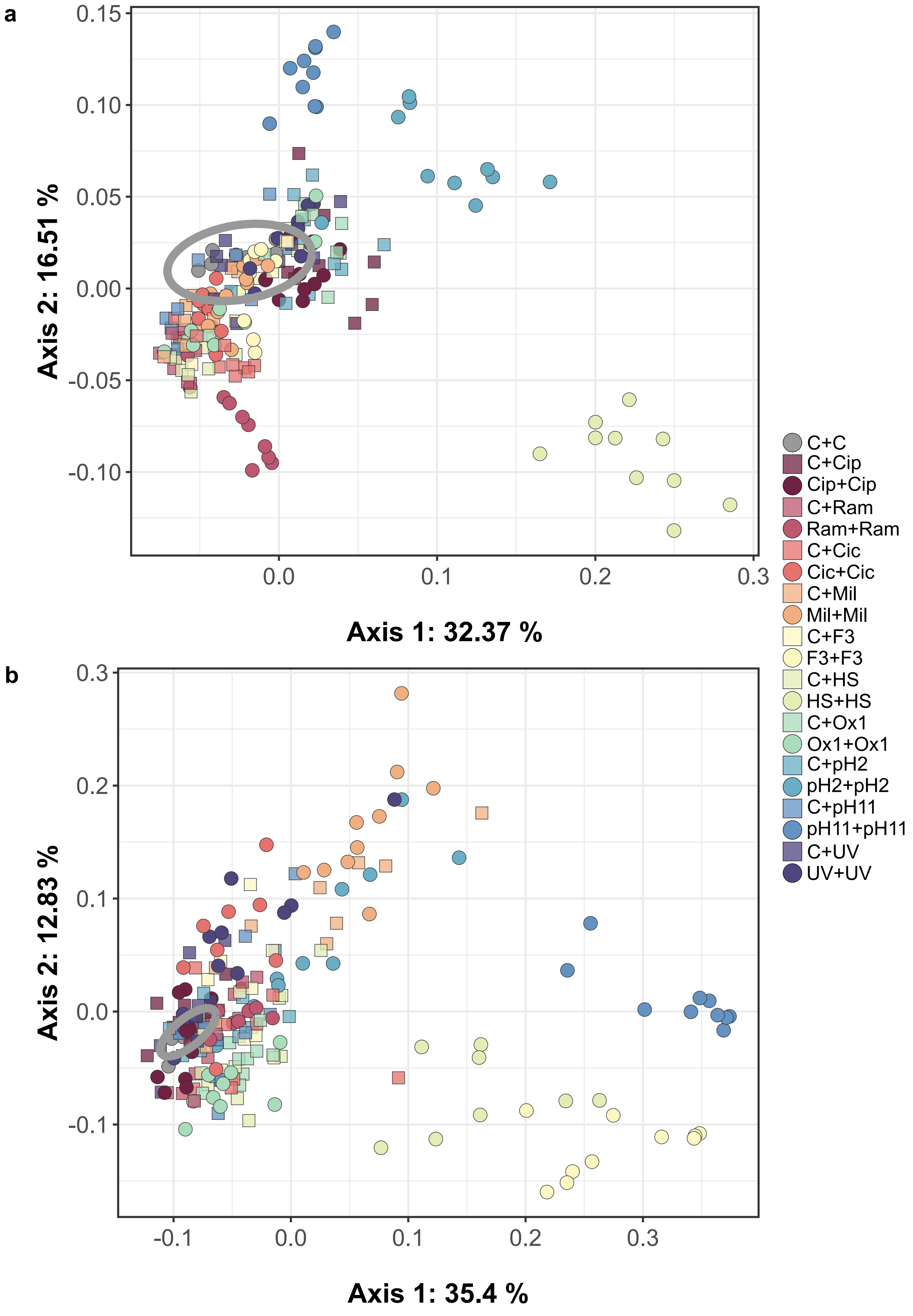

Supplementary Figure 8: Principal coordinate analysis (PCoA) of the prokaryotic (a) and eukaryotic (b) communities, based on the weighted UniFrac distance matrix showing the self-mixed removal treatment, the coalescence treatment and the control samples and the 95% joint confidence ellipse for the control samples.

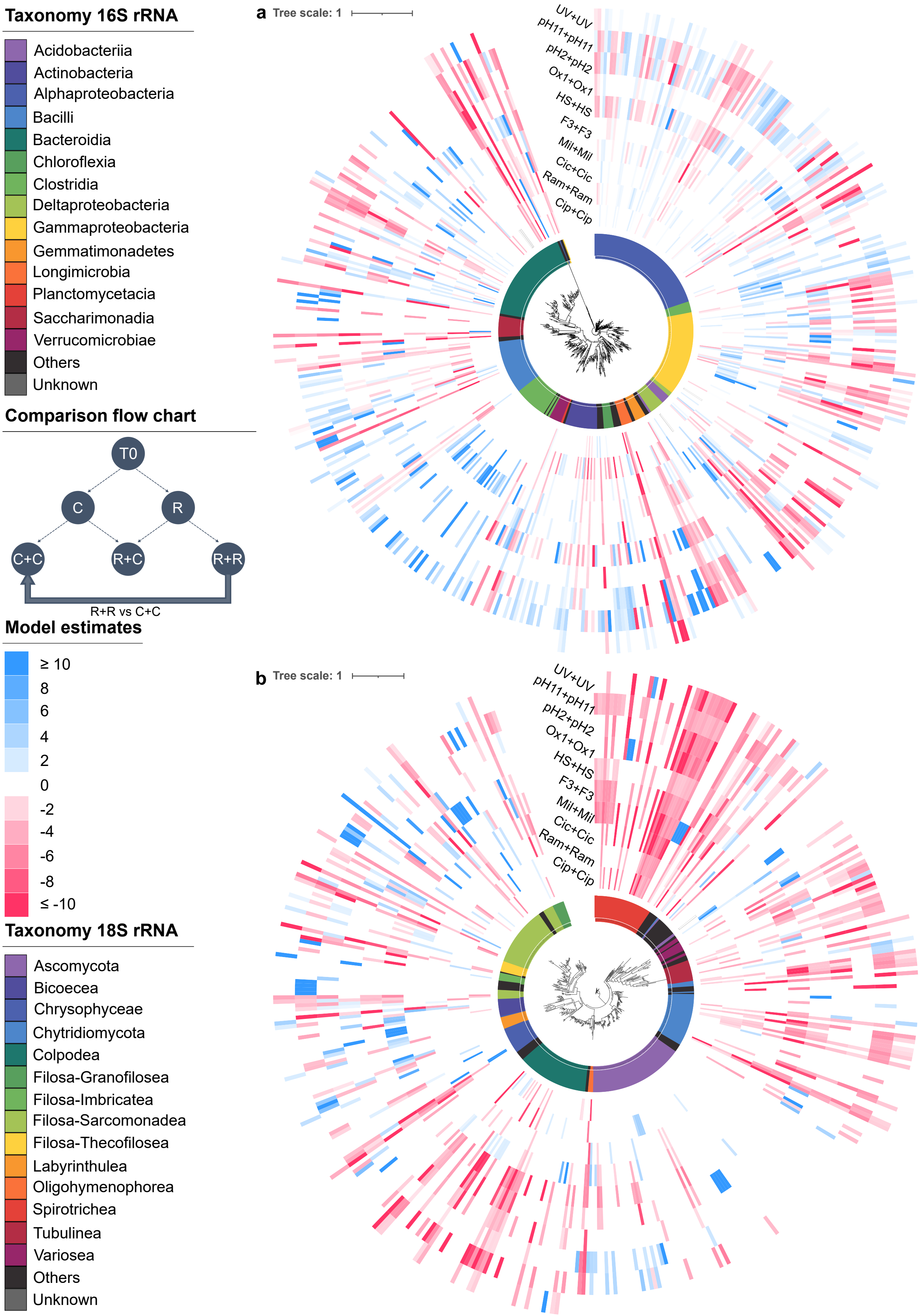

Supplementary Figure 9: Taxonomic relationships and distribution of OTUs significantly affected by the Step 2 removal treatments compared to the Step 2 control. The outer rings show the effect of each self-mixed removal treatment on the relative abundance of the 515 most abundant 16S rRNA OTUs (a) and the 439 most abundant 18S rRNA OTUs (b) compared to the self-mixed control (R+R vs C+C), as estimated using a generalized linear mixed model. The blue and red boxes in the outer rings indicate OTUs with increasing and decreasing fitness respectively, while white boxes indicate OTUs that are not affected by the treatment. The OTU class level is indicated by different colors on the innermost ring.

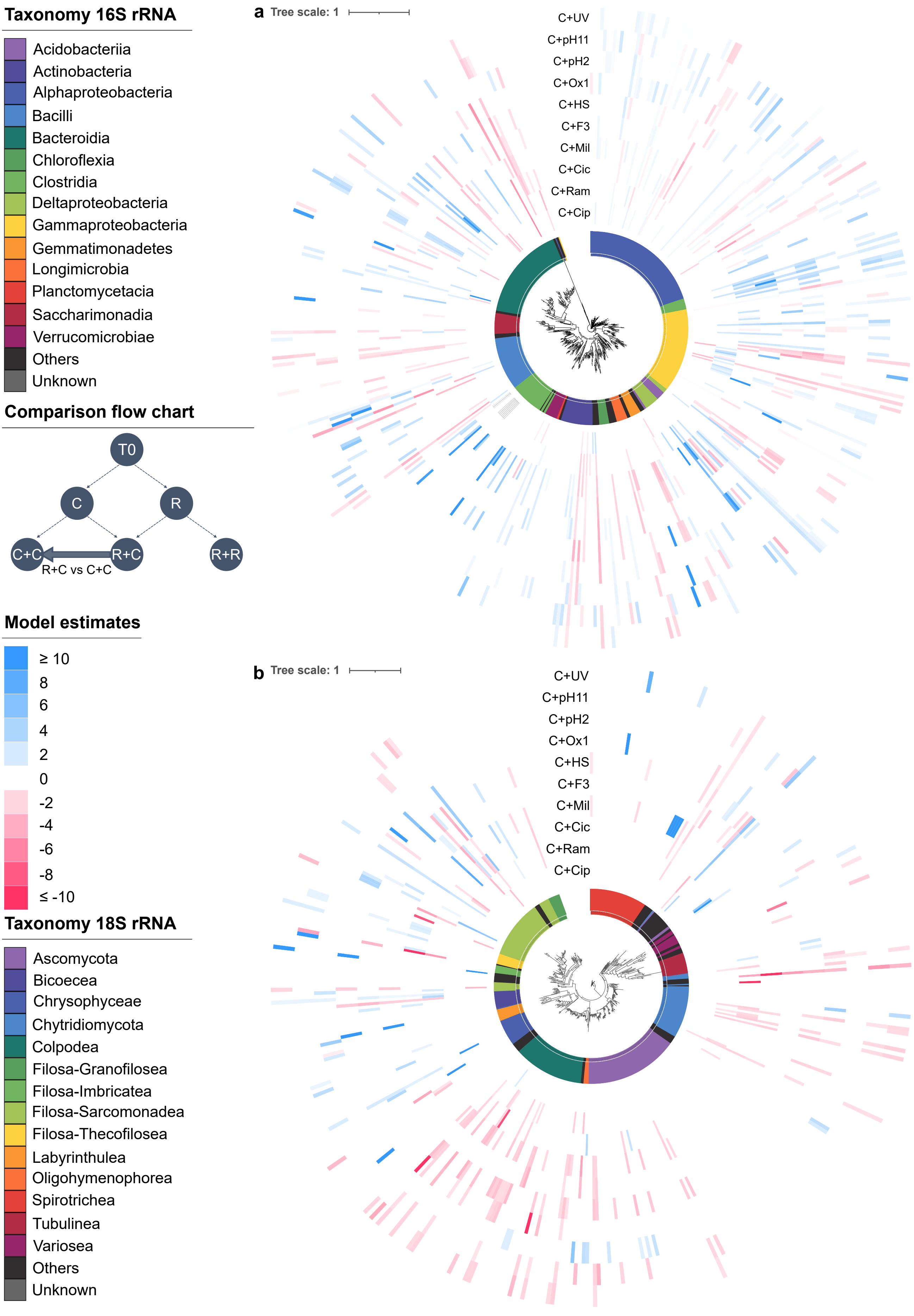

Supplementary Figure 10: Taxonomic relationships and distribution of OTUs significantly affected by the Step 2 coalescence treatments compared to the Step 2 control. The outer rings show the effect of each coalescence treatment on the relative abundance of the 515 most abundant 16S rRNA OTUs (a) and the 439 most abundant 18S rRNA OTUs (b) compared to the self-mixed control (R+C vs C+C), as estimated using a generalized linear mixed model. The blue and red boxes in the outer rings indicate OTUs with increasing and decreasing fitness respectively, while white boxes indicate OTUs that are not affected by the treatment. The OTU class level is indicated by different colors on the innermost ring.

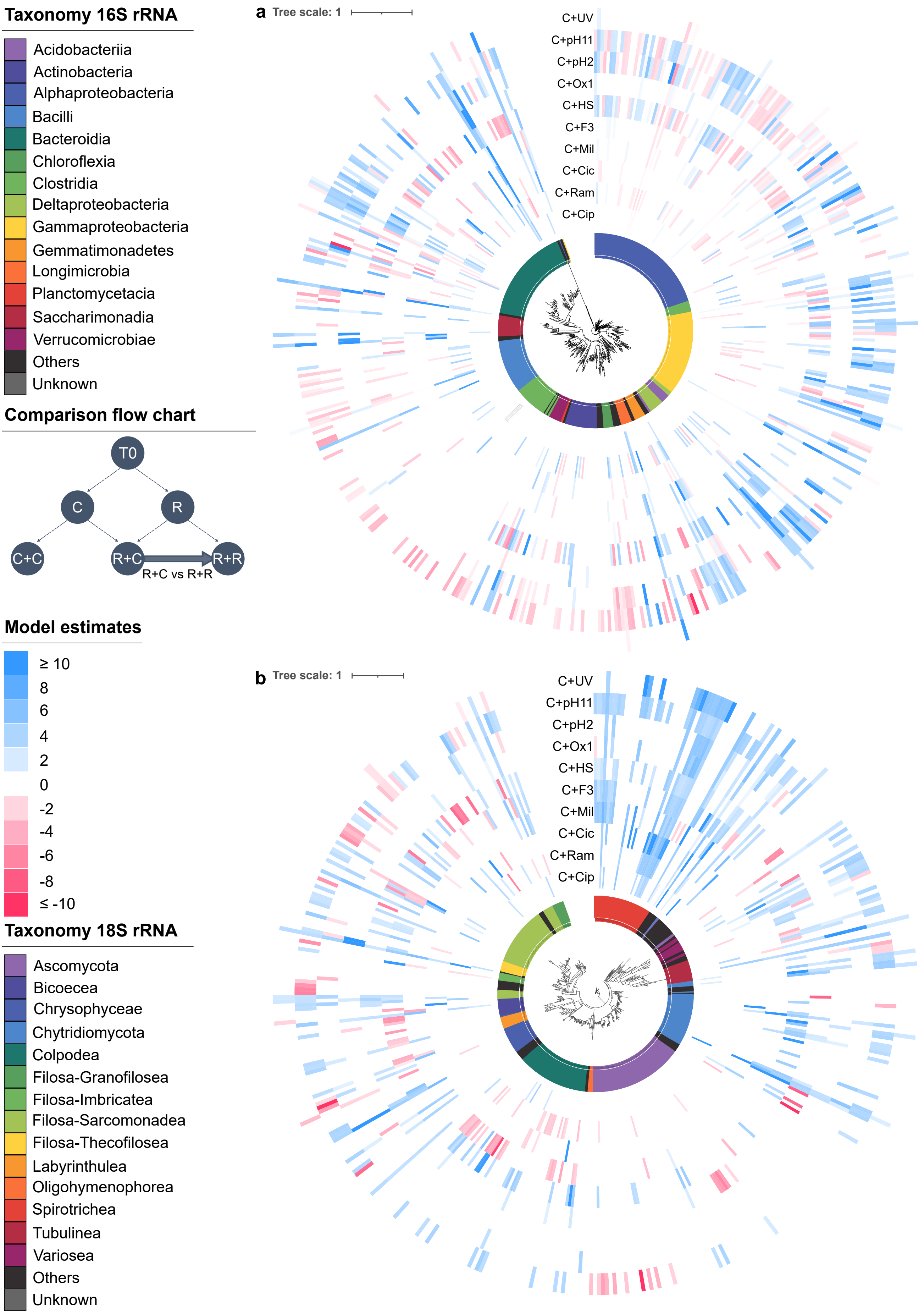

Supplementary Figure 11: Taxonomic relationships and distribution of OTUs significantly affected by the Step 2 coalescence treatments compared to the Step 2 removal treatments. The outer rings show the effect of each coalescence treatment on the relative abundance of the 515 most abundant 16S rRNA OTUs (a) and the 439 most abundant 18S rRNA OTUs (b) compared to its corresponding self-mixed removal treatment (R+C vs R+R), as estimated using a generalized linear mixed model. The blue and red boxes in the outer rings indicate OTUs with increasing and decreasing fitness respectively, while white boxes indicate OTUs that are not affected by the treatment. The OTU class level is indicated by different colors on the innermost ring.

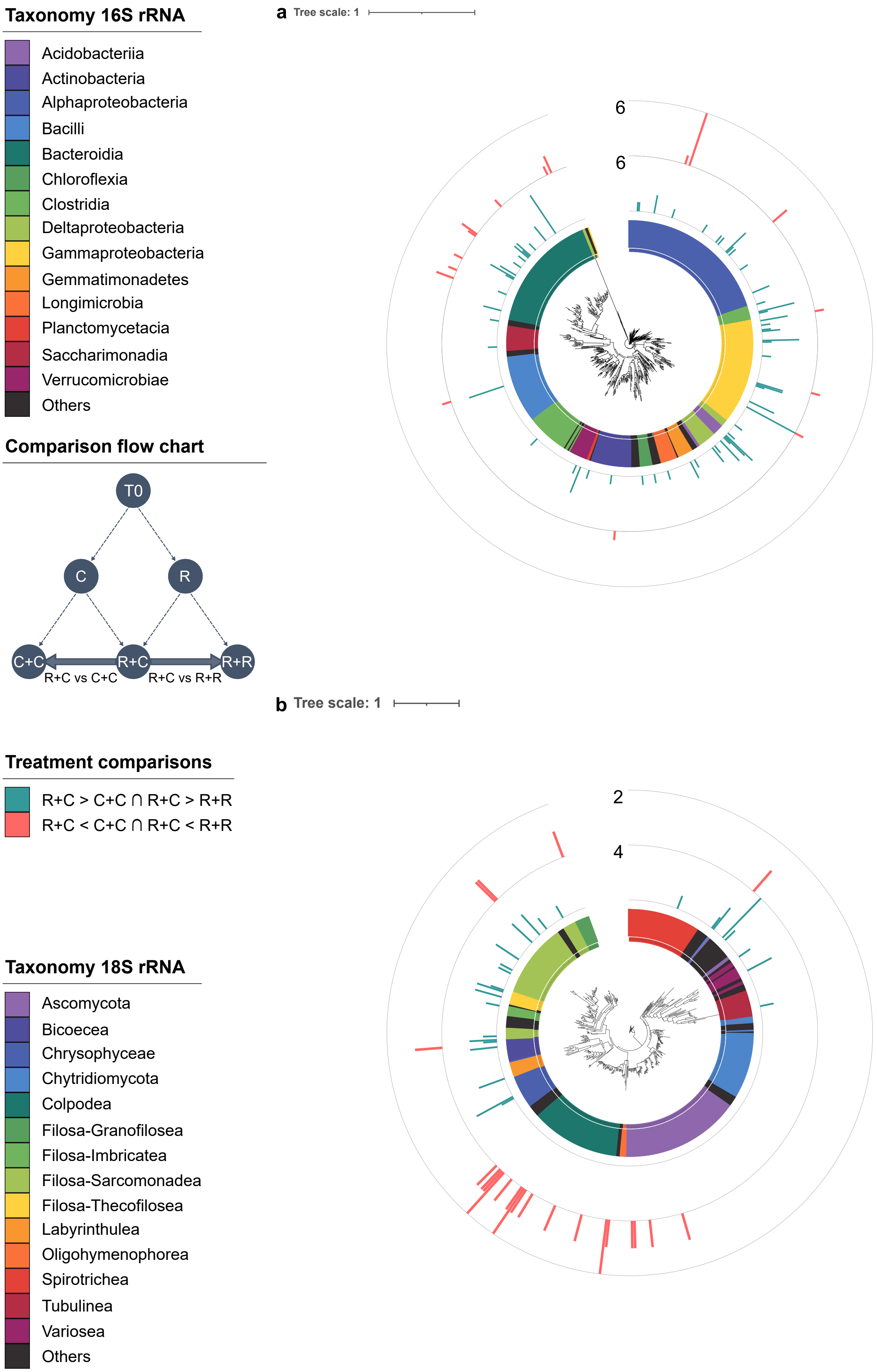

Supplementary Figure 12: Taxonomic relationships and distribution of significantly differentially abundant OTUs across treatments. Outer rings show prokaryotic (a) and eukaryotic (b) OTUs exhibiting relative abundances significantly higher or lower in the coalescence treatments than in the self-mixed source community separately (R+C>C+C ∩ R+C>R+R and R+C<C+C ∩ R+C<R+R). Bar scale is proportional to the number of treatment where the OTU is significantly differentially abundant, with a maximum indicated for each comparison ring. The OTU class is indicated by different colors on the innermost ring.

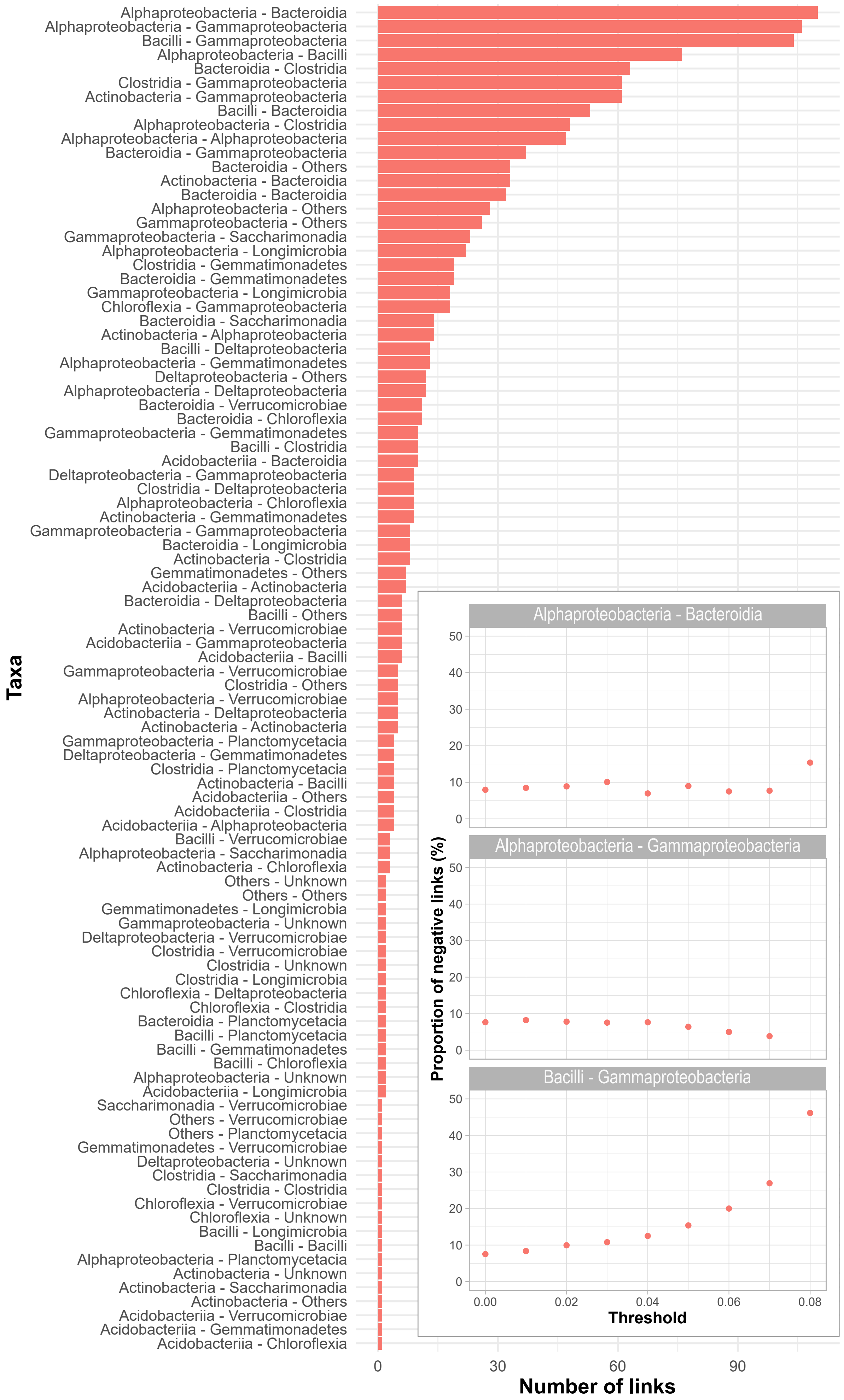

Supplementary Figure 13: Number of negative links between prokaryotic taxa. Number of links represents significant negative partial correlation from the global prokaryotic network inferred from all samples across both experimental steps. The insert represents the proportion of negative links for the top three taxa depending on the strength of the partial correlations

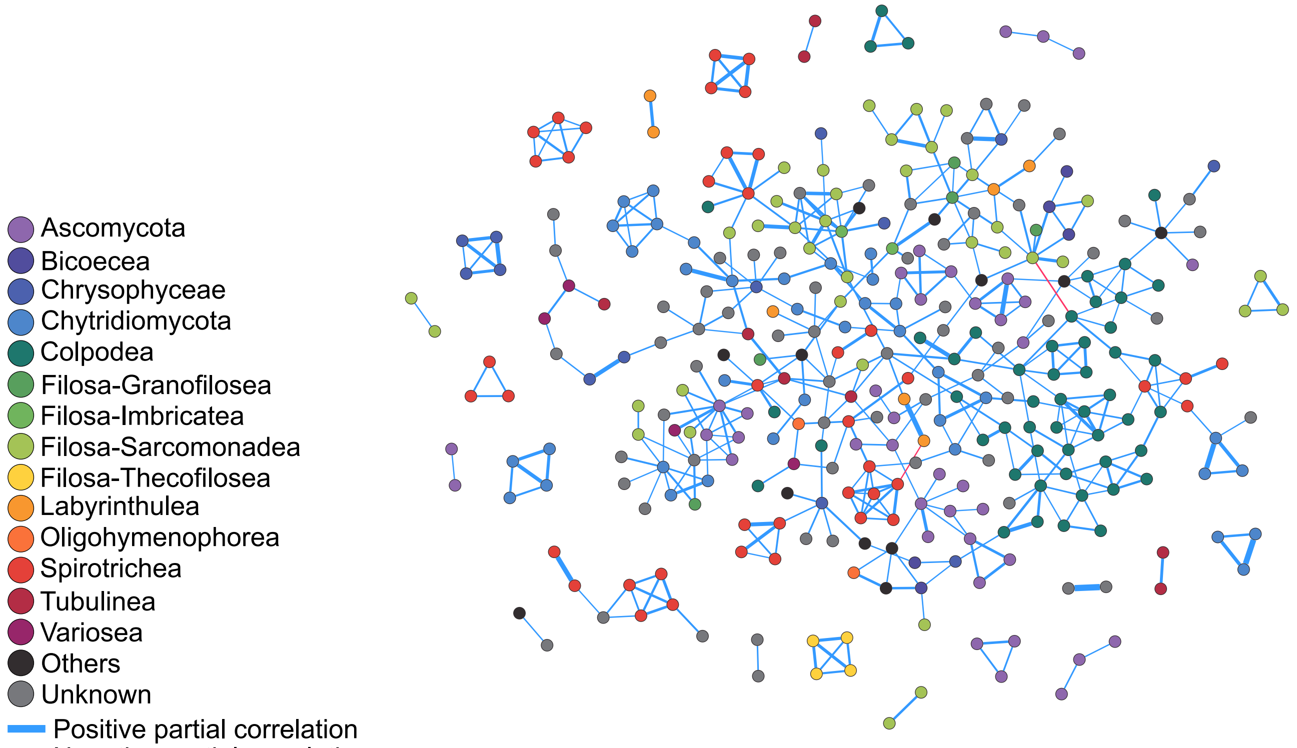

Supplementary Figure 14: Global eukaryotic network inferred from all samples across both experimental steps. Nodes represent OTUs and they are colored according to the OTU taxonomic class. Edges represent partial correlations ρ and they are colored blue if ρ > 0 and red if ρ < 0. Edge width is proportional to |ρ|.

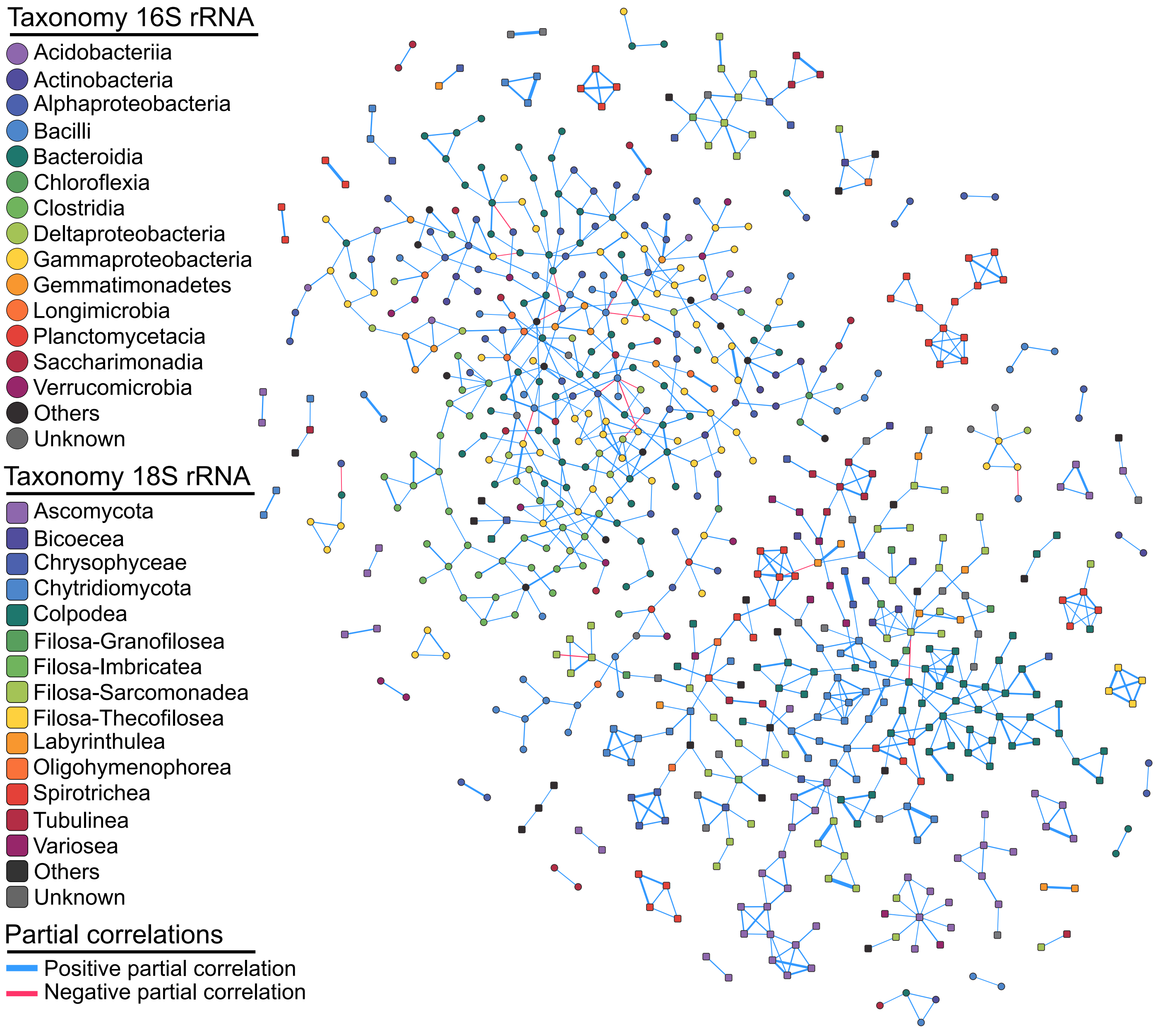

Supplementary Figure 15: Global inter-domain network inferred from all samples across both experimental steps. Nodes represent OTUs and they are colored according to the OTU taxonomic class. Edges represent partial correlations ρ and they are colored blue if ρ > 0 and red if ρ < 0. Edge width is proportional to |ρ|.

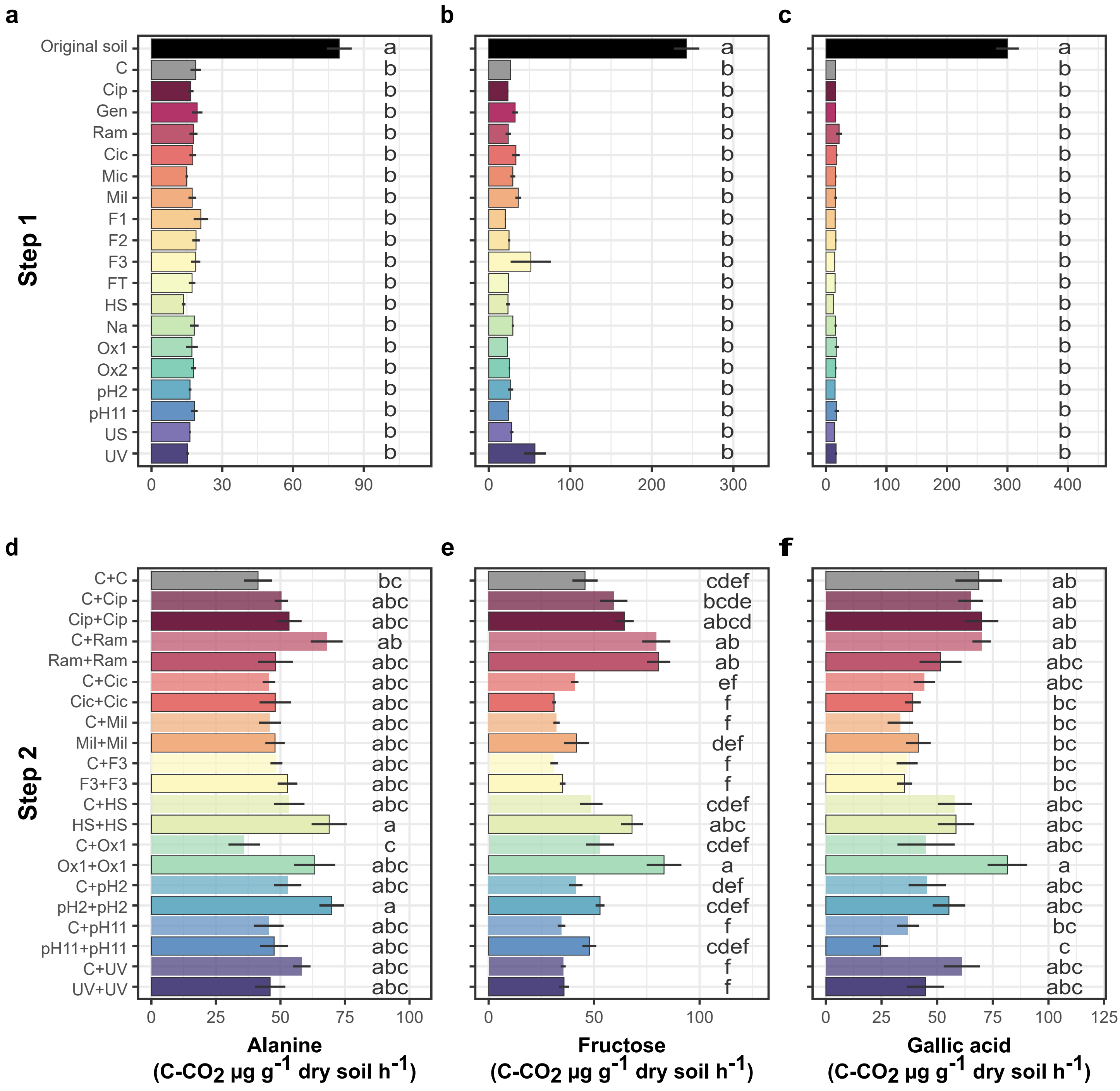

Supplementary Figure 16: Treatment-induced changes in soil respiration rate as proxies of C-cycling. Substrate-induced respiration was measured by the MicroResp™ method using the substrates alanine (a and d), fructose (b and e) and gallic acid (c and f) in the original soil, the removal treatments and the control (Step 1; a, b and c) or in the coalescence treatments and the self-mixed treatments (Step 2; d, e and f) (mean ± s.e.). Letters indicate significantly different statistical groups (Tukey's test, p-value ≤ 0.05).

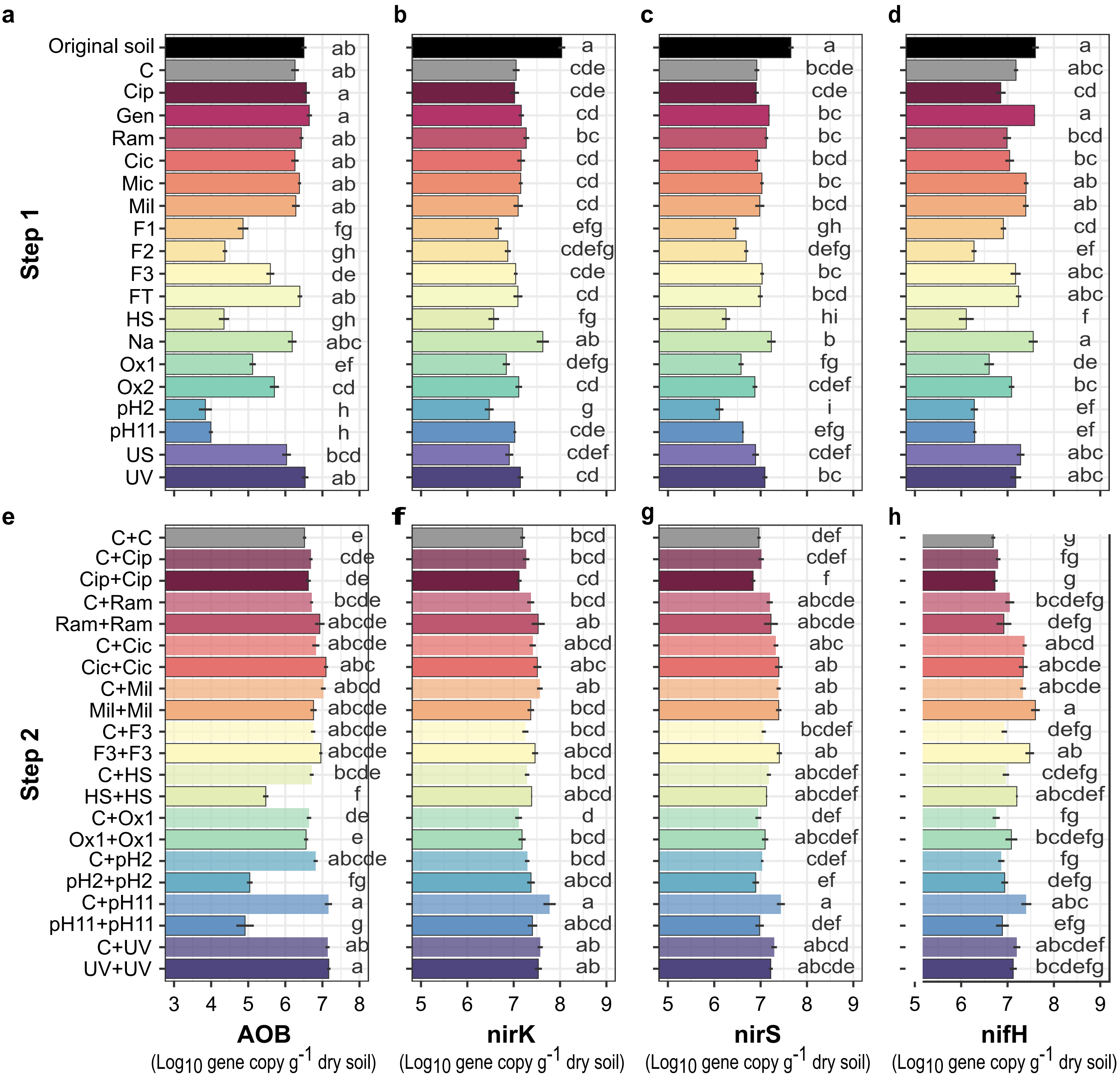

Supplementary Figure 17: Treatment-induced changes in abundance of N-cycle microbial guilds as proxies of N-cycling. Abundances of ammonia-oxidizing bacteria (AOB in a and e), bacterial denitrifiers (nirK in b and f; nirS in c and g) and nitrogen-fixing bacteria (nifH in d and h) in the original soil, the removal treatments and the control (Step 1; a, b, c and d) or in the coalescence treatments and the self-mixed treatments (Step 2; e, f, g and h) (mean ± s.e. of log10-transformed data expressed as gene copy g-1 dry soil). Letters indicate significantly different statistical groups (Tukey's test, p-value ≤ 0.05).
